# Fructose‐Based Single‐Chain Polymer Nanoparticles for GLUT1–Mediated Delivery: Impact of Polymer Design on Uptake and In Vivo Performance

**DOI:** 10.1002/adhm.71345

**Published:** 2026-06-11

**Authors:** Hoang Yen Vo, Linqing Tian, Qiaoyun Wang, Evelyn Szabo, Rebecca Y. Lai, Fariba Dehghani, Martina H. Stenzel

**Affiliations:** ^1^ School of Chemistry University of New South Wales Sydney Australia; ^2^ School of Chemical and Biomolecular Engineering University of Sydney Darlington Australia

**Keywords:** breast cancer, cell uptake, fructose‐based NPs, GLUT transporter, in vivo, Single‐chain nanoparticles

## Abstract

We introduce the preparation and biological evaluation of head‐tail, tadpole‐like, single‐chain nanoparticle (SCNP). These libraries are designed to study the influence of block arrangement on cellular uptake and biodistribution. In the first library, PEG‐NPs consisted of a PEG‐based crosslinked head with a fructose‐containing glycopolymer tail (PEG‐head), while the design was reversed in the second library (Fru‐head). Cellular uptake studies revealed significantly higher uptake of NPs with Fru‐head compared to NPs with PEG head, which is attributed to the surface accessibility of the fructose units to glucose transporters (GLUT). In contrast, the fructose moieties in PEG‐NPs are more likely to be embedded within the core of the PEG head, thereby limiting receptor interaction. Additionally, uptake selectivity followed the order MDA‐MB‐231 > MCF‐7 > RAW 264.7, correlating with GLUT expression levels. Mechanistic studies showed that uptake is strongly suppressed by GLUT1 inhibition but surprisingly unaffected by GLUT5 inhibition, a finding attributed to the pyranose structure of fructose. Fru‐head tadpoles showed prolonged blood circulation and reduced clearance in vivo, with a mean residence time (MRD) of around 21 h, depending on the length of the PEG tail.

## Graphical abstract

Single‐chain nanoparticles have been proposed as drug delivery carriers, but adding a tail can significantly enhance their performance. This was demonstrated using a tadpole‐like structure comprising a head prepared from a UV‐crosslinked fructose‐based glycopolymer capable of targeting GLUT receptors, combined with a PEG‐based tail. Compared with other tested architectures, the tadpole exhibited the highest cell uptake and the longest circulation time.

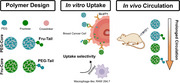

## 1 Introduction

The lack of selectivity of drugs often leads to severe off‐target side effects [1]. Therefore, nanomedicines have been developed to take advantage of the physiological differences between malignant and healthy cells, thereby enhancing the potential for precise targeted delivery [2]. One example would be the heavy reliance of cancer cells on glycolysis for energy production compared with healthy cells. Due to this Warburg effect [3], glucose transporters (GLUTs) are overexpressed in many types of cancer cells [4, 5]. Thus, the use of glycopolymers targeting overexpressed GLUT transporter, has emerged as a promising approach for targeted drug delivery [4, 5].

However, actively targeting tumors with nanoparticles with surface‐bound bioactive functionalities still faces many challenges [6, 7, 8]. Among other obstacles, the formation of the protein corona that covers the bioactive ligands on the surface of the delivery vehicle is one of the culprits that hamper the clinical application [9]. The absorbed proteins, which transform the surface chemistry, play a critical role in the fate of the nanoparticle [10, 11]. Consequently, researchers have tried to understand the correlation between the nanoparticle properties, such as size, charge, and surface chemistry, and the effect on protein absorption and protein composition [12, 13] with the aim of influencing biodistribution and circulation time.

The primary method to control the protein corona is to coat the surface with poly(ethylene glycol) PEG. PEGylation is widely employed to reduce protein adsorption and prolong the circulation time of NPs [14, 15]. While PEGylation effectively mitigates opsonization, the steric hindrance introduced by long‐chain PEG can limit the accessibility of targeting moieties to cell surface receptors, creating the well‐recognized “PEG dilemma” [16, 17]. Therefore, an optimal balance between stealth properties and ligand accessibility requires careful material design [13].

A less exploited pathway to reduce protein absorption is the modulation of the nanoparticle size. There is an agreement in the literature that larger nanoparticles often lead to thicker protein coronas [18, 19]. The interesting aspect is the regime below 10 nm, where nanoparticles are similar in size to proteins, which results in a nanoparticle‐protein complex rather than a closed protein corona [20]. The reduced protein adsorption in smaller nanoparticles reaches a point in dendrimers where the adsorption is relatively limited [21]. Small nanoparticles offer additional advantages, such as improved penetration into tumors [16, 17], and prolonged circulation time [10, 11]. However, very small NPs (< 6 nm) tend to be quickly removed by renal filtration [12, 13].

Single‐chain polymer nanoparticles (SCNPs), which are collapsed polymer chains fixed in a compact, protein‐like structure through intramolecular crosslinking, have recently been investigated for drug delivery [22, 23]. The underlying polymer structure will influence the morphology of the resulting SCNP and, therefore, enable the creation of polymer nanoparticles within the size range of 5–10 nm. We and others have already demonstrated the significant influence of polymer sequence and structure on the in vitro behavior of SCNPs in cancer cells [24, 25, 26]. We hypothesize that the sequence of polymer precursors also plays a pivotal role in governing in vivo interactions. By rationally designing the polymer structure, it should be possible to combine the stealth benefits of PEG with the receptor‐targeting ability of sugar moieties, thereby enhancing both in vitro and in vivo performance.

**FIGURE 1:**
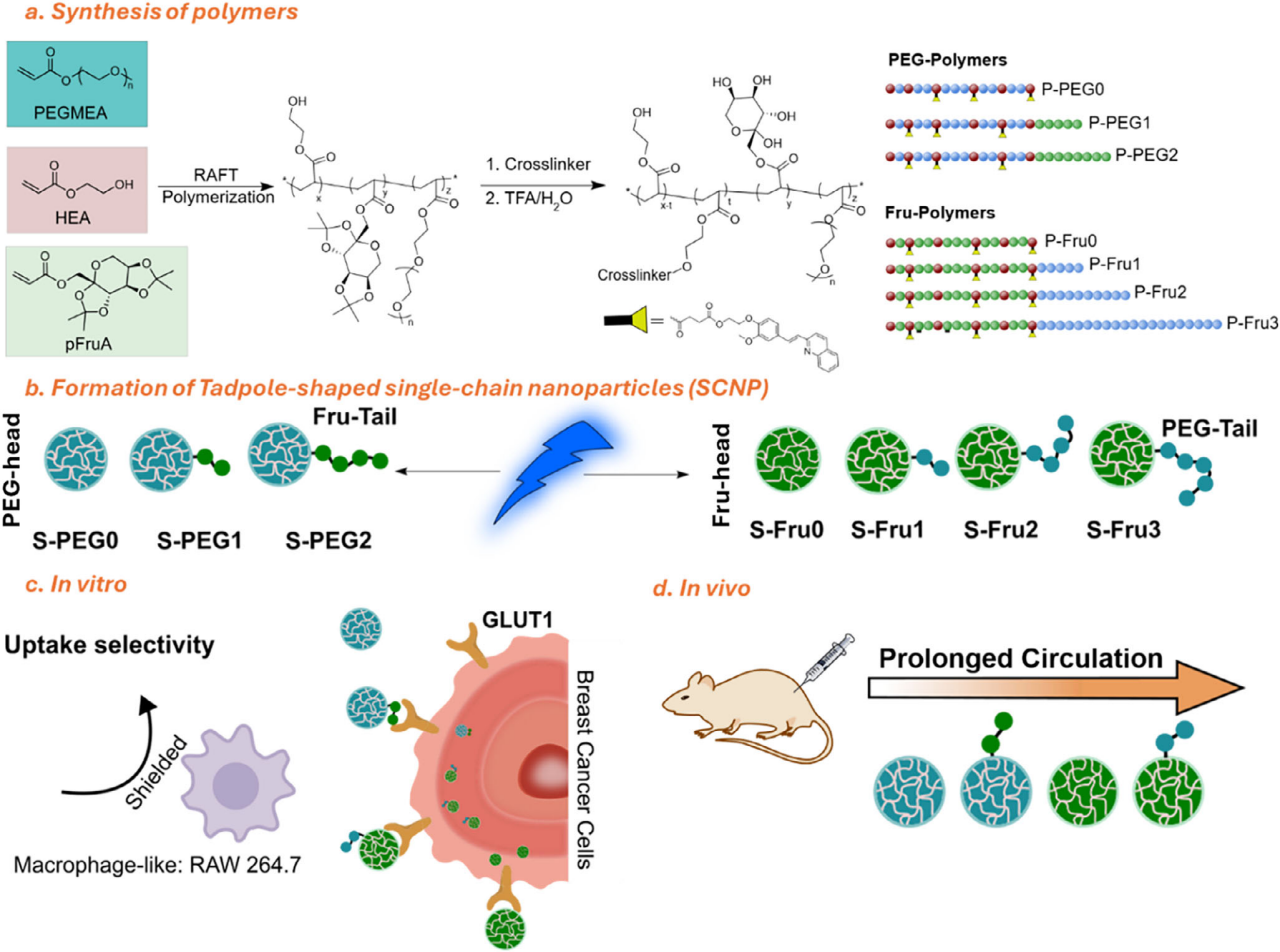
Schematic illustration of the preparation of two libraries of fructose‐based single‐chain polymer nanoparticles (SCNPs) and their in vitro and in vivo biological evaluation. a) Synthesis of PEG‐ and fructose‐polymer libraries via reversible addition‐fragmentation chain transfer (RAFT) polymerization, followed by post‐functionalization with crosslinker and deprotection. b) Formation of two groups of tadpole‐shaped SCNPs with “head‐tail” structure (PEG‐head vs fructose‐head) via blue‐light crosslinking. c) In vitro performance, highlighting structure‐dependent cellular uptake and selectivity mediated by fructose accessibility and interaction with GLUT1 transporters. d) In vivo performance, showing prolonged circulation and architecture‐dependent biodistribution of PEG‐ and fructose‐NPs.

In this study, we designed a library of SCNPs composed of poly(ethylene glycol) methyl ether acrylate (PEGMEA), hydroxyethyl acrylate (HEA), and a fructose‐based monomer (pFruA). We prepared two primary tadpole‐like nanoparticle architectures: “PEG NP” composed of a PEG‐rich head with a fructose tail, and “Fru‐NP,” featuring a fructose‐rich head with a PEG tail. We could demonstrate that the length of the tail is instrumental in optimizing interaction with cells such as GLUT transporter‐overexpressing cells (like MDA‐MB‐231, MCF‐7), and RAW 264.7 macrophage cells, as well as in in vivo behavior (Figure 1). RAW 264.7 cells were included as a model with a different level of GLUT1 expression, and to evaluate the stealth effect of PEG either as a PEG‐rich head in PEG‐NPs or as a PEG tail in Fru‐NPs. Importantly, our results show that the polymer sequence controls both in vitro uptake and in vivo biodistribution and circulation time.

## 2 Results and Discussion

### 2.1 Polymer Synthesis

Protected fructose (pFru) and its monomer (pFruA) were prepared according to previously reported procedures (Scheme S1, Figures S1–S4) [27, 28]. Two polymer libraries were then synthesized via a two‐step RAFT polymerization, each designed with a defined head–tail block copolymer architecture as depicted in Figure 1. Based on our previous studies, reliable SCNP formation is typically achieved at a degree of polymerization (DP) of approximately 100 [24, 29]. Therefore, in this study, we fixed the head at DP approximately 80, while the tail length was systematically varied.

In the first library (PEG‐NP), the head block was composed of a fixed ratio of PEGMEA and HEA, while the tail block contained increasing amounts of pFruA. Specifically, three polymers were synthesized with an increasing fructose tail from pPEG0 to pPEG2. In the second library (Fru‐NP), the head block had a constant ratio of pFruA and HEA, while the PEGMEA tail length was varied to study the effect of PEG shielding (Figure 1). The PEG chain length increased from pFru0 to pFru3. The resulting polymers were characterized by ^1^H NMR in CDCl_3_ (Figures S5–S11).

The crosslinker QIS was synthesized as described in previous works [24, 25], and then coupled to the polymer backbone via an EDC coupling reaction. The resulting polymer‐crosslinker conjugates were characterized by ^1^H NMR in CDCl_3_ (Figure S12–S18) and size exclusion chromatography (SEC) in DMF (Figure S19), with molecular weights summarized in Table S1. The conjugates were named P‐X, where X denotes the polymer backbone (e.g., P‐pPEG1, P‐pPEG2, P‐pFru0). Following crosslinker conjugation, most polymers exhibited minimal changes in SEC retention times. However, slight shifts toward shorter retention times were observed for P‐Fru0 and P‐Fru1, possibly indicating modest increases in molecular weight. Additionally, a pronounced shoulder at shorter retention times in some samples suggests the occurrence of interchain crosslinking during the coupling step.

The protecting groups on fructose moieties were removed using a trifluoroacetic acid‐water (TFA/H_2_O) mixture (FigureS20–S25). The resulting polymers were named by removing the “p” prefix (e.g., P‐Fru0, P‐PEG1).

### 2.2 SCNPs Preparation and Characterization

**FIGURE 2:**
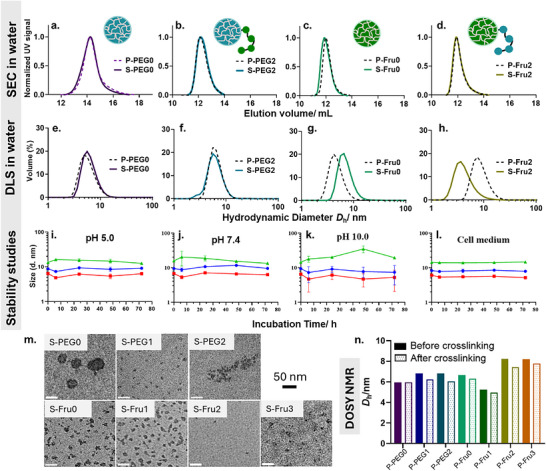
Characterization of polymers before and after crosslinking. a‐d) Aqueous size‐exclusion chromatography (SEC) traces of polymers before (black dashed) and after (colored) crosslinking. e‐h) Hydrodynamic diameter of samples before (black dashed) and after (colored) crosslinking, collected from dynamic light scattering (volume) at 1 mg mL^−1^ in water. i–l) Colloidal stability of S‐Fru2 NP under different pH and biological conditions, polymer nanoparticles (1 mg mL^−1^) incubated in (i) MES (2‐(N‐morpholino)ethanesulfonic acid) buffer (50 mm, pH 5.0), (j) phosphate buffered saline (PBS) buffer (50 mm, pH 7.4), (k) carbonate buffer (50 mm, pH 10.0), and (l) cell culture medium. For cell medium conditions, 0.5 mL of polymer solution (2 mg mL^−1^ in water) was mixed with 0.5 mL 1× DMEM supplemented with 10% FBS, 1% penicillin, and 1% Glutamax. Samples were incubated at 37°C and analyzed after 0, 6, 24, 48, and 72 h. The error bars represent the standard error of the mean (SEM) (n = 3). Blue, red, and green traces correspond to volume‐, number‐, and Z‐average size distributions, respectively. m) Transmission electron microscopy (TEM) images of crosslinked NPs, scale bar 50 nm. n) Hydrodynamic diameter of samples, calculated from diffusion‐ordered spectroscopy (DOSY) NMR before and after crosslinking at 5 mg mL^−1^ in D_2_O.

The crosslinked SCNPs, which are now coined as S‐X, were prepared by the intramolecular collapse of the polymer dissolved in water, followed by blue light irradiation to fix the compact structure. Similar to our previous study, the crosslinking conversion was stopped at approximately 50% conversion for better cellular uptake [25]. This was achieved by following the UV–Vis signal (Table S2 and Figure S26). The crosslinked SCNPs were characterized using various techniques, as shown in Figure 2, including SEC (Figure 2a–d and Figure S27) and DLS (Figure 2e–h and Figure S28) measurements. The SEC traces remained unimodal with no significant aggregation or interchain crosslinking after collapse (Figure 2a–d and Figure S27). In contrast, the hydrodynamic diameter as determined by DLS (Figure 2e–h and Figure S28) was reduced after crosslinking in the case of Fru‐NPs (except S‐Fru0), while PEG‐NPs exhibited no significant size change. One reason might be that intramolecular crosslinking stretches the PEG chains to compensate for crowding.

Further characterization is required to confirm the formation of SCNPs. Accordingly, we conducted diffusion‐ordered spectroscopy (DOSY) NMR in D_2_O (Figure 2n and Table S3). The results showed that all crosslinked polymers, except P‐PEG0, had smaller hydrodynamic diameters after crosslinking, with sizes below 10 nm. Transmission electron microscopy (TEM) images of dried SCNPs from aqueous solution (Figure 2m) supported the formation of small NPs, although the limited resolution and the tendency of small NPs to aggregate in dry conditions hampered the analysis. Most SCNPs appeared as spherical particles with diameters under 20 nm, except for S‐PEG0 (*d* = 51.2 nm), which formed larger aggregates in the dry state. These nanoparticles also demonstrated good colloidal stability (Figure 2i–l and Figure S29). S‐Fru2, chosen as a representative sample, exhibited good colloidal stability across different pH values and in DMEM for up to 3 days (Figure 2i–l). Consistently, all nanoparticles displayed stable size distributions in full DMEM during short‐term incubation (Figure S29).

### 2.3 In Vitro Studies

#### 2.3.1 Cytotoxicity and Cellular Uptake

The in vitro biocompatibility of these delivery platforms was evaluated on two breast cancer cell lines, MCF‐7 and MDA‐MB‐231 (Figure S30). No cytotoxicity was observed in both cell lines, even at 1000 µg mL^−1^ after 3 days.

**FIGURE 3:**
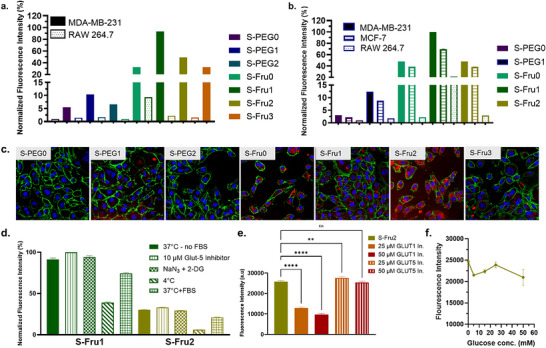
Biological evaluation of polymer NPs on different cell lines. a.) Comparison of cellular uptakes of all crosslinked NPs in MDA‐MB‐231 and RAW 264.7 cells. Cells were incubated with NPs at 5 µm for 4 h; the error bars represent SEM (n = 3). b.) Comparison of cellular uptakes of some crosslinked NPs in MDA‐MB‐231, MCF‐7, and RAW 264.7 cells. Cells were incubated with NPs at 2.5 µm for 4 h; the error bars represent SEM (n = 3). c.) CLSM images of MDA‐MB‐231 cells, incubated with crosslinked NPs at 2.5 µm for 4 h. Cy5 dye was conjugated to NPs, cells’ nuclei were stained blue with Hoechst dye, and the membranes were stained green with wheat germ agglutinin (WGA); the error bars represent SEM (n = 3). d.) Uptake mechanism study at different conditions on MDA‐MB‐231 cells, using S‐Fru1 and S‐Fru2. Cells were incubated with NPs at 2.5 µm for 4 h in serum‐free DMEM, except for 37°C+FBS samples; the error bars represent SEM (n = 3). e.) Uptake with GLUT1 and GLUT5 inhibitors at different inhibitor concentrations on MDA‐MB‐231 cells. Cells were preincubated with the inhibitor for 1 h before loading NP (2.5 µm for 2 h); the error bars represent SEM (n = 3). Statistical significance was determined using ordinary one‐way ANOVA (^*^ p < 0.05, ^**^ *p* < 0.01, ^***^ *p* < 0.001, ^****^ *p* < 0.0001). f.) The glucose competition uptake study on MDA‐MB‐231 cells. Cells were preincubated in glucose‐free DMEM medium overnight before being incubated with serum‐free medium with different glucose concentrations (0, 5, 15, 25, 50 µm) for 1 h. S‐Fru2 was added to each sample at 2.5 µm and incubated for another 2 h; the error bars represent SEM (n = 3).

The cellular uptake was evaluated in breast cancer cells (MDA‐MB‐231 and MCF‐7) and RAW 264.7 macrophage‐like cells (Figure 3a,b). These cell lines were selected due to their differences in GLUT1 and GLUT5 transporter expression. Breast cancer cells are known to overexpress glucose transporters at varying levels [30, 31, 32]. RAW 264.7 cells have low levels of GLUT1 and GLUT3 transporters, but concentration is significantly upregulated in activated cells during inflammation. GLUT5 is, in contrast, barely expressed on RAW 264.7 cells [33]. Notably, Fru‐NPs showed significantly higher uptake in all cell lines compared to PEG‐NPs (Figure 3a,b). In PEG‐NPs, increasing the fructose tail length slightly improved uptake (from S‐PEG0 to S‐PEG1), but this method was generally ineffective (lower uptake at S‐PEG2). This may be because the longer fructose chains become embedded within the PEG‐rich head during crosslinking, which limits their accessibility to binding to the GLUT transporters on cell membranes.

In contrast, Fru‐NPs displayed a clear structure‐activity relationship. The PEG tail, being more hydrophilic, likely remained solvated and extended into the aqueous phase, allowing the fructose‐rich head to stay exposed for GLUT transporter interaction. As the PEG chain length increased from S‐Fru0 to S‐Fru1, uptake significantly improved, which can be attributed to better solubility and dispersion of the Fru‐NPs. However, further extension of the PEG chain to 50 units (S‐Fru2) or 108 units (S‐Fru3) led to a marked decrease in uptake (from 49% to 32.4% in MDA‐MB‐231 cells, Figure 3a). This is consistent with the known shielding effect of PEG [34]. Long PEG chains can hinder ligand–receptor interactions by steric repulsion or by covering the fructose moieties.

Confocal microscopy images of MDA‐MB‐231 cells (Figure 3c) supported these trends, showing stronger fluorescence signals for Fru‐NPs compared to PEG‐NPs. Additionally, SCNPs containing shorter PEG chains (S‐Fru1 and S‐Fru2) displayed more pronounced intracellular localization than long‐chain PEG (S‐Fru3), which may be due to shielding of fructose when the PEG‐based block reached a critical length. This supports the hypothesis that nanoparticle design critically influences internalization efficiency.

#### 2.3.2 Uptake Mechanism

To understand the mechanism behind the uptake of our NPs, a series of inhibition studies were conducted using MDA‐MB‐231 cells. Flow cytometry and confocal imaging demonstrated the highest nanoparticle uptake in MDA‐MB‐231 cells, followed by MCF‐7 and RAW 264.7 cells (Figure 3b). Sodium azide (NaN_3_), a metabolic inhibitor, and 2‐deoxy‐D‐glucose (2‐DG), glucose analog that inhibits glycolysis, are used together to inhibit cellular uptake because they target different energy metabolisms and can suppress cellular energy‐dependent processes. However, these metabolic inhibitors did not result in a reduced uptake (Figure 3d), suggesting that an energy‐independent pathway takes up these SCNPs. We showed recently that these ultrasmall NPs can enter the cells by penetrating through the cell membrane, enabling direct cytosolic delivery of nanoparticles [35], using processes such as membrane fusion. A moderate decrease in uptake (30–50%) was observed at 4°C, which may suggest that a fraction of nanoparticles was taken up by an energy‐dependent process. However, it needs to be considered that cooling the solution may have several effects that might have led to reduced uptake. The solubility of glycopolymers is dependent on the temperature, where lowering the temperature has the potential to result in clustering, changing the NP structure [36]. In another study using mannose‐based compounds, it was found that binding to macrophage‐bound mannose receptors was, in fact, reduced at lower temperatures [37]. Moreover, basic studies on the interaction between GLUT transporters and various carbohydrates have shown that low temperatures reduce the internal molecular movement of the GLUT transporter, as the membrane is in a gel phase [38]. Because of these unknowns, we tested the role of GLUT transporters by GLUT1 and GLUT5 inhibition (Figure 3e). Surprisingly, while GLUT5 inhibition had no impact on nanoparticle uptake, GLUT1 inhibition significantly suppressed uptake by approximately 50% at 25 µm and 60% at an inhibitor concentration of 50 µm (Figure 3e). The remaining 40–50% of nanoparticle uptake is likely mediated by GLUT‐independent pathways, including nonspecific endocytic processes or passive transport mechanisms. This interpretation is supported by our observation that NP internalization and uptake is not entirely energy‐dependent, as evidenced by reduced yet detectable uptake at 4°C and in the presence of 2‐DG, which inhibits active metabolic activity. This contrasts expectations that fructose should engage with GLUT5, not GLUT1. Here, we need to understand the specificity of GLUT transporters. Among the 14 different known GLUT transporters, only GLUT5 is specific to fructose. GLUT5 can transport fructose in its pyranose and furanose forms, although studies with fluorescent derivatives of both forms showed higher uptake of the furanose form by MCF‐7 cells [39]. In this bespoke study, the authors found that other GLUT transporters, such as the exemplified GLUT2, are indiscriminatory toward the various sugar forms [39]. It is therefore feasible that our polymer, where fructose is attached in pyranose form, has a relatively low affinity to GLUT5 and may be more internalised by other GLUT transporters. Interestingly, glucose competition assays (0–50 mm) (Figure 3f) showed no significant differences, hence the NPs may bind only at a distinct or allosteric site on GLUT1, or utilize a non‐competitive transport pathway. These findings agree with the uptake selectivity observed across the three cell lines (Figure 3a,b), where MDA‐MB‐231 cells express higher levels of GLUT1 than MCF‐7 [30, 31, 32], while RAW 264.7 cells show only normal expression [40].

**FIGURE 4:**
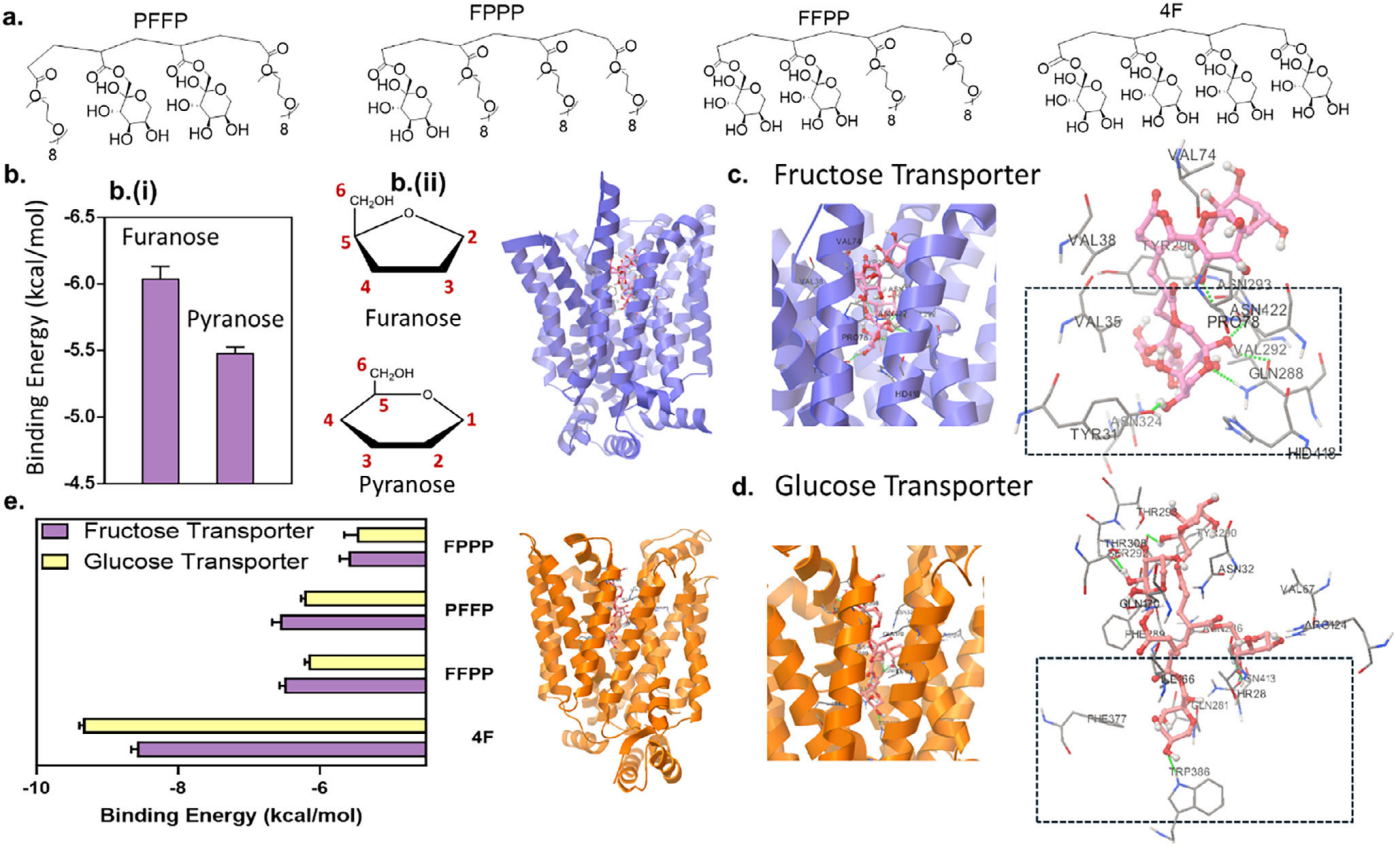
Molecular docking studies of the binding interactions between outward‐open fructose/glucose transporter and various polymer models. a.) Structure of fructose or fructose‐PEG models. b.) The binding energy (i) between the fructose transporter (GLUT5) and fructopyranose or fructofuranose (ii). The bindings between the polymer model 4F and the fructose (c.) or glucose (d.) transporter are visualized, showing the overall protein‐polymer complex, a zoomed‐in view of the binding pocket, and detailed hydrogen bonding interactions, respectively. Hydrogen bonds are represented by green dashed lines. e.) Plot of binding energies between polymer models and the fructose/glucose transporter. Additional pyranose and furanose fructose structures, as well as docking results for the PFFP, FPPP, and FFPP models with fructose and glucose transporters, are provided in Figure S31.

To further investigate the mechanism underlying the structure‐dependent uptake, molecular docking simulations were performed using fructose‐based polymer models composed of four fructose units (4F). Structural variants were generated by replacing two or three fructose units with PEG moieties positioned either at the center (FPPF), at both ends (PFFP), or in three consecutive positions (FPPP), to evaluate how PEG incorporation affects binding affinity toward the fructose and glucose transporters (Figure 4A). In solution, fructose exists primarily in either the pyranose (6‐membered ring) or furanose (5‐membered ring) form (Figure 4Bii), with the furanose conformer exhibiting stronger binding to the GLUT5 (Figure 4Bi). Comparison of the binding energies across models (Figure 4E) indicates that the flexibility and accessibility of fructose pendants play a key role in optimizing interactions with membrane transporters. Among all models, 4F showed the strongest binding to both transporters, with higher affinity for the glucose transporter than the fructose one, where one fructose unit was inserted into the glucose binding pocket and formed multiple hydrogen bonds with surrounding amino acids (Figure 4C,D) [41]. Incorporation of PEG segments progressively weakened the overall binding affinity, as PEG does not directly interact with the protein surface.

In the glucose transporter (Figure 4D), the FFPP and PFFP models could still occupy the binding pocket, indicating that the polymer remained accessible for recognition. In contrast, in the FPPP model, the PEG chains shielded the fructose units and occupied the binding site, preventing GLUT transporter interaction.

For the GLUT5 (Figure 4C), PEG substitution introduced steric hindrance that shifted FFPP and PFFP away from the fructose binding pocket. Although the binding energies of these PEG‐containing models were comparable for both types of receptors, their recognition outcomes differed, with preferable polymer and glucose transporter interactions.

Overall, these results demonstrate that fructose in the pyranose form exhibits a higher affinity toward glucose transporter than the fructose one in the pure fructose (FFFF) model, while for PEG‐containing polymers, transporter recognition is governed by fructose accessibility and polymer composition rather than binding energy alone. Maintaining accessible, flexible fructose pendants is essential for effective transporter recognition and cellular uptake

These findings highlight the importance of fine‐tuning polymer architecture for optimized nanoparticle performance. In this study, short‐to‐medium PEG chains (S‐Fru1 and S‐Fru2) appear to provide a balance between solubility and GLUT transporter accessibility, leading to improved cellular uptake. Besides structure‐dependent uptake, we also observed significant uptake selectivity in three cell lines (Figure 3a,b), with significantly higher uptake observed in breast cancer cells than in RAW 264.7 cells. This selectivity for cancer cells is more likely driven by the stealth effect and the receptor‐mediated uptake pathway, which is more pronounced in breast cancer cells [30, 31, 32].

### 2.4 In Vivo Pharmacokinetic and Biodistribution Studies

Our previous study revealed the effect of fructose length on the bioactivity of glycopolymer, where longer chain length showed higher cellular uptake, better penetration in spheroids, and reduced undesirable uptake by the mononuclear phagocyte system (MPS) in vivo [42]. Building upon this foundation, the present work extends this concept by examining how both fructose chain length and the head–tail positioning of PEG *vs* fructose within SCNPs collectively govern their pharmacokinetic and biodistribution behaviors in vivo.

**FIGURE 5:**
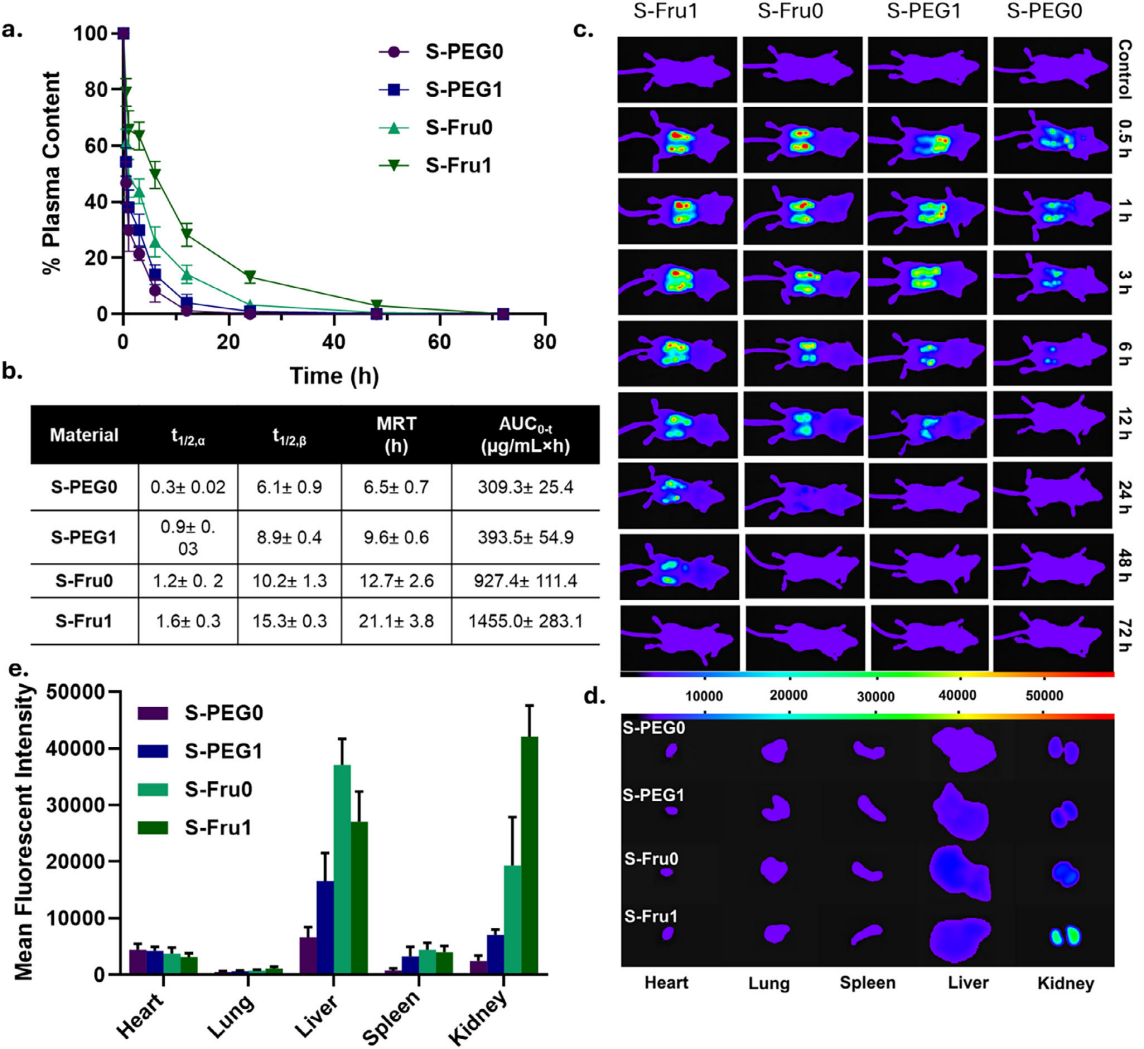
Pharmacokinetic and Biodistribution of Cy5‐labeled crosslinked NPs in healthy BALB/c mice. a.) Time‐dependent plasma concentration of PEG‐NPs and Fru‐NPs, obtained by quantifying fluorescence intensity in plasma samples (n = 5) collected at different time points (0.5, 1, 3, 6, 12, 24, 48, and 72 h post‐injection). b.) Pharmacokinetic parameters for PEG‐NPs and Fru‐NPs, calculated using a two‐compartment model. c.) in vivo fluorescence imaging of mice treated with Cy5‐labelled NPs at different time points up to 72 h using IVIS after IV administration. d) ex vivo fluorescence images of major organs at 72 h post‐injection of PEG‐NPs and Fru‐NPs. e) Mean fluorescent intensity of major organs at 72 h post‐injection of PEG‐NPs and Fru‐NPs. Error bars represent standard deviation (n = 5).

The in vivo pharmacokinetics of SCNPs were evaluated in healthy mice following intravenous injection. Plasma concentrations relative to the initial dose are shown in Figure 5a, and the corresponding pharmacokinetic parameters, derived from a two‐compartment model, are summarized in Figure 5b. All four SCNPs exhibited a rapid distribution of half‐life (t_1/2,α_), followed by a slower elimination from the blood circulation with a long elimination half‐life (t_1/2,β_). In the absence of targeting moieties, S‐PEG0 showed rapid clearance and was quickly eliminated (t_1/2,β_ = 6.1 h). Interestingly, the introduction of a small fructose tail (S‐PEG1) markedly prolonged circulation (t_1/2,β_ = 8.9 h), suggesting that surface‐exposed fructose alters nanoparticle–biological interactions in vivo, thereby transiently delaying clearance pathways. In contrast, Fru‐NPs exhibited remarkable blood retention and prolonged circulation time. Even without PEG, S‐Fru0 circulated longer than S‐PEG1, and addition of a short PEG tail in S‐Fru1 further increased t_1/2β_ (15.3 h) and nearly doubled the MRT (21.1 h) and AUC (1455.0 µg/mL × h) (Figure 5b).

Whole‐body fluorescence imaging corroborated these findings (Figure 5c). Without fructose, the fluorescence signal from S‐PEG0 faded within 6 h post‐injection, whereas the addition of a short fructose tail in S‐PEG1 remained detectable up to 12 h. In contrast, Fru‐NPs maintained strong fluorescence throughout 72 h, consistent with their prolonged circulation. Ex vivo analysis of major organs at 72 h post‐injection (Figure 5d,e) revealed architecture‐dependent biodistribution. PEG‐NPs (S‐PEG0, S‐PEG1) are primarily accumulated in the liver, whereas Fru‐NPs are distributed across both the liver and kidneys. Introducing fructose into PEG‐NPs (S‐PEG1 vs. S‐PEG0) increased hepatic uptake, consistent with enhanced biological interactions under physiological conditions. Conversely, the addition of PEG to Fru‐NPs (S‐Fru1 vs. S‐Fru0) decreased liver accumulation, in line with the stealth effect of PEG that mitigated opsonization while retaining accessible fructose for potential receptor interactions.

Overall, these data highlight how balancing PEG shielding with surface‐exposed fructose allows precise control of NPs' fate in vivo. Among all formulations, S‐Fru1 achieved the most favorable performance, prolonged circulation, moderated hepatic uptake, and persistent fluorescence for 72 h, offering a clear design principle for the development of fructose‐functionalized SCNPs with optimized pharmacokinetics and biodistribution profiles under physiological conditions.

When comparing in vitro and in vivo results, we observe that the fructose tadpole S‐Fru1 is superior in many respects, as it improves blood circulation and increases uptake by cancer cells. Of course, in vitro and in vivo studies cannot be directly compared, as we compare a very complex living system with cells grown in a dish in cell culture media that contain proteins from different sources [43]. The order S‐Fru1> S‐Fru0> S‐PEG1> S‐PEG0 might therefore be only coincidental. We might have to look at some subtleties in the biodistribution. It is notable that both Fru‐based materials exhibit greater accumulation in the liver and spleen, suggesting increased uptake by the mononuclear phagocyte system (MPS). Closer analysis of the uptake by RAW264.7 (Figure 3a) mirrors the in vivo work. The high accumulation of S‐Fru1 and S‐Fru0 is not surprising considering the high expression of GLUT transporters in the liver [44]. This would contradict the longer circulation time of S‐Fru1 and S‐Fru0. However, the organ distribution was recorded after 72 h only. At this time point, most PEG particles have been eliminated from the bloodstream, and they may also have already been cleared by the liver and kidneys. The intensity distribution over time does suggest this (Figure 5c). It is therefore not clear why clearance of the fructose‐based nanoparticles is slower.

## 3 Conclusions

In this study, we developed two types of single‐chain polymer nanoparticles with similar size and stability but different head–tail arrangements. The results showed that the structural difference significantly affects their in vitro uptake and in vivo performance. Fru‐NPs, with fructose in the head and a PEG tail on the surface, showed much higher uptake than PEG‐NPs, consistent with improved accessibility of the fructose groups for interactions with glucose transporters. Uptake selectivity was observed in three cell lines (MDA‐MB‐231, MCF‐7, and RAW 264.7), depending on the level of GLUT1 expression. Mechanistic studies suggest that in vitro uptake happened through a passive, glucose‐transporter‐associated pathway. Interestingly, polymer architecture and head–tail block arrangement were strongly associated with differences in in vivo circulation and biodistribution. Fru‐NPs showed prolonged circulation time compared to PEG‐NPs. Overall, these findings demonstrate that the arrangement of blocks in SCNPs has a significant influence on their performance in vitro and in vivo. These findings underscore the importance of polymer architecture in glucose‐transporter‐associated delivery under physiological conditions.

## 4 Experimental Section

### 4.1 Materials

D‐fructose, triethylamine (TEA), poly(ethylene glycol) methyl ether acrylate (PEGMEA), 2‐hydroxyethyl acrylate (HEA), azobisisobutyronitrile (AIBN), *N, N*‐dimethylformamide (DMF), 4‐dimethylaminopyridine (DMAP), *N*‐(3‐dimethylaminopropyl)‐N′‐ethylcarbodiimide hydrochloride (EDC.HCl), trifluoroacetic acid (TFA), and trioxane were purchased from Sigma–Aldrich. Sulfuric acid, sodium hydroxide (NaOH), dichloromethane (DCM), ethyl acetate, *n*‐hexane, and diethyl ether were purchased from Chem Supply. MCF‐7 (CODE: 86012803) and MDA‐MB‐231 (CODE: 92020424) cells were purchased from CellBank Australia. RAW 264.7 cell line murine (UNSPSC Code: 41106514) was purchased from Merck.

### 4.2 Preparation of SCNPs

The fructose‐based monomer (pFruA) was synthesized according to a previously reported procedure [45], with the detailed protocol provided in the Scheme S1, Figures S1–S4. The RAFT agent (M‐CPP) and crosslinker (QIS) were also prepared following published methods [24].

#### 4.2.1 Synthesis of Polymer pFru0

In a 25 mL round‐bottom flask, pFruA (3.3 g, 10.44 mmol), HEA (0.78 g, 6.7 mmol), M‐CPP (0.058 g, 0.2 mmol), and AIBN (0.0065 g, 0.04 mmol) were dissolved in DMF (10 mL). Next, trioxane (0.1 g, 1.11 mmol) was added as an internal standard. The ratio of pFruA:HEA:M‐CPP:AIBN was 52:34:1:0.2. The solution was degassed by bubbling with N_2_ for 30 min before reacting at 70°C for 24 h. The crude was collected and precipitated in cold ether and dried under a vacuum to yield a yellow solid. The monomer conversion was >92% for pFruA and >93% for HEA by ^1^H NMR.

Detailed synthetic procedures for other polymers are provided in the Supporting Information.

#### 4.2.2 Conjugation of the Crosslinker

The crosslinker was conjugated to the polymer via an EDC coupling. In a typical procedure, polymer pFru1 (1.1847 g, 0.039 mmol), QIS (0.1636 g, 0.39 mmol), and DMAP (0.071 g, 0.58 mmol) were dissolved in DCM (10 mL), followed by dropwise addition of EDC·HCl (0.2232 g, 1.16 mmol in 2 mL DCM). The mixture was purged with N_2_ and stirred overnight at room temperature. The product was collected, dialyzed against MeOH for 3 days using a dialysis tube with MWCO 3.5 kDa. The solvent was evaporated at reduced pressure, and the polymer was dried in a vacuum.

#### 4.2.3 Deprotection of Fructose

The fructose moieties were deprotected by stirring with a trifluoroacetic acid (TFA)/H_2_O mixture. The detailed procedure is reported in the Supporting Information.

#### 4.2.4 SCNP Preparation

The crosslinked SCNPs were prepared by employing the [2+2] cycloaddition reaction of the crosslinkers in water, under blue light irradiation. Detailed optimization conditions for all polymers are reported in, Table S2 and Figure S26.

### 4.3 Molecular Docking Method

The crystal structure of outward‐open fructose transporter (GLUT5) (PDB id: 4YBQ) and outward‐open glucose transporter (PDB id: 4ZWC) were taken from the protein data bank [41, 46]. Protonation states were assigned using the Protein Preparation Tool and subsequently used for molecular docking studies [47]. Molecular docking was carried out with the Autodock Vina package to study the binding between fructopyranose/fructofuranose and the GLUT5 [48]. The binding between various polymer models and the fructose/glucose transporters was also studied. Four polymer models were designed to evaluate the impact of PEG incorporation on fructose polymer–transporter binding. The fructose polymer model consisting of 4 repeat units of fructose (FFFF) was first constructed. Next, two fructose units were replaced with PEG chains either on the side (FFPP) or at the ends (PFFP). Another model, FPPP, was made by replacing three fructose units with PEG chains (Figure 4). These four simplified oligomer models (FFFF, FFPP, PFFP, and FPPP) were selected to represent distinct local environments with varying degrees of PEG‐induced shielding. By systematically varying the position of fructose units (terminal vs. internal), the models enabled evaluation of how steric hindrance influences transporter binding. A four‐unit length was chosen as the minimal sequence capable of capturing these accessibility‐dependent effects while maintaining computational efficiency. Polar hydrogens and partial atomic charges were added, while non‐polar hydrogens were merged for all dockings. For each model, the dockings were repeated five times with an exhaustiveness value of 20. For each docking run, the binding energies of the top three conformations were recorded. Among the five docking repetitions, the top five binding energies were selected to calculate the average binding performance. Additionally, the top binding conformations were visualized and presented in Figure 4.

### 4.4 Cell and Animal Studies

#### 4.4.1 In Vitro Cell Studies

Cell studies were carried out using two breast cancer cell lines (MCF‐7 and MDA‐MB‐231) and a macrophage cell line (RAW‐264.7). The Dulbecco's Modified Eagle Medium (DMEM) High Glucose with 10% Fetal Bovine Serum (FBS), 1% Glutamax, and 1% Penicillin was used as a cell medium. Cells were cultured on tissue culture flasks in a 5% CO_2_ incubator at 37°C.

#### 4.4.2 In Vitro Cell Viability

The in vitro cytotoxicity of NPs was assessed by using the sulforhodamine B (SRB) assay. Cells were seeded into 96‐well plates at a concentration of 4000 cells/well and 9000 cells/well for MCF‐7 and MDA‐MB‐231, respectively, and cultured for 2 days at 37°C with 5% CO_2_. Subsequently, polymer solutions at various concentrations (1000 to 6.25 µg mL^−1^) were added and incubated for 3 days. Next, cells were fixed with 10% trichloroacetic acid (TCA) solution before staining with SRB solution. After staining, 200 µL of a 10 mm Tris buffer solution was added to each well, and the absorbance at 490 or 570 nm was measured using a Bio‐Rad BenchMark microplate reader.

#### 4.4.3 Analysis of Cell Uptake by Flow Cytometry

Cellular uptake of Cy5‐labeled SCNPs by MCF‐7, MDA‐MB‐231, and RAW 264.7 cells was quantified using flow cytometry (BD FACSymphony A3).

For MCF‐7 vs. RAW 264.7 comparison, cells (5×10^5^/well) were seeded in 6‐well plates and cultured for 2 days at 37°C, 5% CO_2,_ before incubation with 2.5 µm SCNPs (triplicate wells) for 4 h. For the MDA‐MB‐231 vs. RAW 264.7 comparison, cells were incubated with 5 µm SCNPs. After incubation, cells were detached with trypsin/EDTA, neutralized with DMEM, collected by centrifugation, and resuspended in cold HBSS.

Cells were initially gated based on FSC‐A versus SSC‐A to exclude debris, followed by singlet discrimination using FSC‐A versus FSC‐H to remove doublets. Cy5 fluorescence was quantified within the gated singlet population.

Fluorescence from 10 000 events per well was detected using a 660/20 nm band‐pass filter, and median fluorescence intensity (MFI) values were calculated from triplicate samples using FlowJo software. Fluorescence intensities of the polymer stock solutions were measured and used to normalize the data accordingly.

#### 4.4.4 Analysis of Cell Uptake by Confocal Laser Scanning Microscopy (CLSM)

Qualitative uptake of Cy5‐labeled SCNPs on MDA‐MB‐231 cells was carried out by using laser scanning confocal microscopy (Leica SP8 DLS, equipped with a 63.0×1.40NA oil immersion objective). MDA‐MB‐231 cells (1.5 × 10^5^/dish) were cultured in 35 mm FluoroDishes for 2 days, at 37°C, 5% CO_2_. Cells were then incubated with SCNPs (working concentration 2.5 µm) for 2 h. After incubation, cells were washed with HBSS, fixed with 4% paraformaldehyde, and stained with Hoechst dye and Wheat Germ Agglutinin (WGA) before imaging. ImageJ software was used for image processing.

#### 4.4.5 In Vivo Pharmacokinetic and Biodistribution Study

The pharmacokinetic and biodistribution studies were conducted at the University of New South Wales, in accordance with the University of New South Wales Animal Ethics Committee Guidelines (UNSW Clearance number iRECS5988). Six‐week‐old female BALB/c mice were purchased from Australian BioResources.

To evaluate blood circulation, a total of 25 healthy female BALB/c mice were divided into five groups (n = 5 per group) and intravenously injected with Cy5‐labeled samples (1 mg/kg Cy5). For pharmacokinetics, 20 µL of blood was collected at 0.5, 1, 3, 6, 12, 24, 48, and 72 h, centrifuged to obtain plasma, and Cy5 fluorescence was quantified. Pharmacokinetic parameters were calculated using a two‐compartment model.

For biodistribution, procedures matched the pharmacokinetic study to ensure consistency. One mouse received PBS to provide a background signal. Whole‐body optical imaging was performed at each time point using an IVIS Lumina II system (650 nm excitation, 670 nm emission). After 72 h, mice were euthanized, and major organs were harvested, washed with saline, and imaged ex vivo to assess tissue accumulation.

## Author Contributions

Hoang Yen Vo: Conceptualization, Methodology, Investigation, Data curation, Formal analysis, Cell culture, Cytotoxicity assays, Writing – original draft, Writing – review & editing, Visualization. Linqing Tian: Investigation, Methodology, Molecular docking, Writing – review & editing. Qiaoyun Wang: Flow cytometry, Confocal imaging, animal studies. Evelyn Szabo: Flow cytometry. Rebecca Y. Lai: TEM analysis, review & editing. Fariba Dehghani: Review & editing. Martina H. Stenzel: Supervision, Resources, Writing – review & editing, Visualization, Project administration.

## Conflicts of Interest

The authors declare no conflicts of interest.

## Supporting information




**Supporting File**: adhm71345‐sup‐0001‐SuppMat.pdf.

## Data Availability

The data that support the fundings of this study are available from the corresponding author upon reasonable request.
