# Supplementary material for: Fructose‐Based Single‐Chain Polymer Nanoparticles for GLUT1–Mediated Delivery: Impact of Polymer Design on Uptake and In Vivo Performance
Source: Adv Healthc Mater. 2026 Jun 11;15(27):e71345. doi: 10.1002/adhm.71345 (PMC13378471; doi:10.1002/adhm.71345)
Supplement: Supplementary file 1 — Supporting File: adhm71345‐sup‐0001‐SuppMat.pdf. [file ADHM-15-0-s001.pdf]

ELECTRONIC SUPPORTING INFORMATION

Fructose-Based Single-Chain Polymer Nanoparticles for GLUT1–Mediated Delivery: Impact of Polymer Design on Uptake and *in vivo* Performance

Hoang Yen Vo[1†], Linqing Tian[1], Qiaoyun Wang[1], Evelyn Szabo[1], Rebecca Y. Lai[1], Fariba Dehghani[2], Martina H. Stenzel[1]*

[1]School of Chemistry, University of New South Wales, Sydney, New South Wales 2052, Australia

[2]School of Chemical and Biomolecular Engineering, University of Sydney, Darlington, New South Wales 2008, Australia

†now at School of Chemical and Biomolecular Engineering, University of Sydney, Darlington, New South Wales 2008, Australia

KEYWORDS. Single-chain nanoparticles, fructose-based NPs, GLUT transporter, cell uptake, *in vivo*, breast cancer.

Contents

List of figures 3

List of tables 4

List of scheme 4

1. Analytical instruments 5

   a. Size Exclusion Chromatography 5

   b. Nuclear Magnetic Resonance Spectroscopy (NMR) 5

   c. UV-Visible Spectroscopy (UV-Vis) 5

   d. Dynamic Light Scattering (DLS) 5

   e. Transmission Electron Microscope (TEM) 6

   f. Diffusion-Ordered NMR Spectroscopy (DOSY-NMR) 6

2. Synthesis 7

   a. Synthesis of RAFT agent (M-CPP) 7

   b. Synthesis of polymer backbones 11

   c. Synthesis of the crosslinker 16

   d. Conjugation of the crosslinker 16

   e. Deprotection of fructose 22

   f. SCNP preparation 26

   g. Coupling of Cy5-COOH 30

References 32

List of figures

Figure S1. ^{1}H NMR spectrum of pFru in DMSO-d_6....... 8
Figure S2. ^{13}C NMR spectrum of pFru in DMSO-d_6....... 9
Figure S3. ^{1}H NMR spectrum of pFruA in $CDCl_3$. 10
Figure S4. ^{13}C NMR spectrum of pFruA in $CDCl_3$. 10
Figure S5. ^{1}H NMR spectrum of PEG0 in $CDCl_3$ 13
Figure S6. ^{1}H NMR spectrum of pPEG1 in $CDCl_3$ 13
Figure S7. ^{1}H NMR spectrum of pPEG2 in $CDCl_3$ 14
Figure S8. ^{1}H NMR spectrum of pFru0 in $CDCl_3$ 14
Figure S9. ^{1}H NMR spectrum of pFru1 in $CDCl_3$ 15
Figure S10. ^{1}H NMR spectrum of pFru2 in $CDCl_3$ 16
Figure S11. ^{1}H NMR spectrum of pFru3 in $CDCl_3$ 16
Figure S12. ^{1}H NMR spectrum of P-PEG0 in $CDCl_3$....... 17
Figure S13. ^{1}H NMR spectrum of P-pPEG1 in $CDCl_3$....... 18
Figure S14. ^{1}H NMR spectrum of P-pPEG2 in $CDCl_3$....... 18
Figure S15. ^{1}H NMR spectrum of P-pFru0 in $CDCl_3$....... 19
Figure S16. ^{1}H NMR spectrum of P-pFru1 in $CDCl_3$....... 19
Figure S17. ^{1}H NMR spectrum of P-pFru2 in $CDCl_3$....... 20
Figure S18. ^{1}H NMR spectrum of P-pFru3 in $CDCl_3$....... 20
Figure S19. SEC traces in DMF of the polymer backbones (a and b) and their polymer-crosslinker conjugates (c and d). 21
Figure S20. ^{1}H NMR spectra of copolymer in $CDCl_3$ before (P-pPEG1, upper) and after (P-PEG1, lower) deprotection. 23
Figure S21. ^{1}H NMR spectra of copolymer in $CDCl_3$ before (P-pPEG2, upper) and after (P-PEG2, lower) deprotection. 23
Figure S22. ^{1}H NMR spectra of copolymer in $CDCl_3$ before (P-pFru0, upper) and in DMSO-d_6 after (P-Fru0, lower) deprotection. 24
Figure S23. ^{1}H NMR spectra of copolymer in $CDCl_3$ before (P-pFru1, upper) and in DMSO-d_6 after (P-Fru1, lower) deprotection. 24
Figure S24. ^{1}H NMR spectra of the copolymer in $CDCl_3$ before (P-pFru2, upper) and in DMSO-d_6 after (P-Fru2, lower) deprotection. 25

Figure S25. ^1H NMR spectra of the copolymer in $CDCl_3$ before (P-pFru3, upper) and in DMSO-d_6 after (P-Fru3, lower) deprotection. 25
Figure S26. UV-Vis of polymer solution at 1 mg mL^{-1} in water, after different irradiation times. 27
Figure S27. SEC traces in aqueous solution (Milli Q water + NaN_3) of the PEG-NPs (a-c) and Fru-NPs (d-g) before (blue) and after (red) crosslinking. * in 40% MeCN, 0.1% TFA in water. 28
Figure S28. DLS measurements of polymer samples before (blue) and after (red) crosslinking. 28
Figure S29. Cytotoxicity test of the polymer NPs on MCF-7(a) and MDA-MB-231 (b) cells after 72 hours of incubation at various polymer concentrations. 30
Figure S30. Mean fluorescent intensity of major organs at 72 hours post-injection of PEG-NPs and Fru-NPs. Error bars represent standard deviation (n = 5). ....**Error! Bookmark not defined.**

List of tables

Table S1. Molecular weight analysis of the polymer backbones and their corresponding conjugates with the crosslinker. 21
Table S2. Optimization condition. Polymers were dissolved in water at a concentration of 1 mg mL^{-1}. 26
Table S3. Diffusion coefficients and hydrodynamic diameter of polymer before (P-X) and after (S-X) crosslinking. Polymer concentration at 5 mg mL^{-1} in D_2O 29

List of scheme

Scheme S1. Preparation of 2,3:4,5-di-O-isopropylidene-β-D-fructopyranose (pFru) 7

1. Analytical instruments

a. Size Exclusion Chromatography

Size exclusion chromatography (SEC) in DMF was carried out on a Shimadzu Prominence modular SEC system equipped with an LC-20AD pump, RID-20A refractive index detector, SIL-20A HT autosampler, and a guard column. Separation was achieved using three Phenomenex columns (pore sizes 10^3, 10^4, and 10^5 Å), with HPLC-grade DMF, with 0.05% w/v BHT and 0.01% w/v LiBr as the eluent. The system was operated at 0.8 mL/min and 50°C using a CTO-20A oven, with an injection volume of 25 µL. Molecular weight calibration was performed using commercial polymethyl methacrylate (PMMA) standards.

For aqueous SEC, measurements were performed on an Agilent 1260 Infinity HPLC system with a quaternary pump. Milli-Q water with NaN_3 was used as the eluent at 1.0 mL/min. For P-PEG0, the eluent was Milli-Q water with 40 vol% acetonitrile (MeCN) and 0.1 vol% trifluoroacetic acid.

b. Nuclear Magnetic Resonance Spectroscopy (NMR)

Nuclear Magnetic Resonance (NMR) spectroscopy was used to characterize the structure of the synthesized compounds and to determine polymerization conversion. Both ^1H and ^{13}C NMR spectra were recorded on a Bruker Advance III 400 MHz spectrometer with an integrated autosampler. Spectra were processed and analyzed using MestReNova software. Depending on sample solubility, $CDCl_3$, DMSO-d_6 were used as solvents for NMR measurements.

c. UV-Visible Spectroscopy (UV-Vis)

UV-Visible (UV-Vis) spectroscopy was employed to monitor the dimerization reaction during the crosslinking step. Measurements were performed on a VARIAN Bio Cary 60 UV-Vis spectrophotometer coupled with a Perkin Elmer Differential Scanning Calorimeter, covering a wavelength range of 200–800 nm and absorbance below 5 Abs. Data were collected over the visible range (400–800 nm) at 25°C.

d. Dynamic Light Scattering (DLS)

Dynamic light scattering (DLS) measurements were performed using a Malvern Zetasizer Nano ZS with a He–Ne laser (633 nm, 5 mW) at a scattering angle of 173°. Samples were prepared in

Milli-Q water at various concentrations and filtered through a 0.45 µm filter to remove dust. Data were analyzed using Malvern Zetasizer Software version 6.20

e. Transmission Electron Microscope (TEM)

Transmission electron microscopy (TEM) images were acquired on a JEOL 1400 microscope equipped with a dispersive X-ray analyzer and a Gatan CCD camera at 80 kV. Samples were prepared by casting the micellar solution onto copper grids, air-dried, and negatively stained with uranyl acetate. Particle size distributions were determined using ImageJ, analyzing 100 particles per sample.

f. Diffusion-Ordered NMR Spectroscopy (DOSY-NMR)

Diffusion-Ordered NMR Spectroscopy (DOSY-NMR) was employed to measure the diffusion coefficients of linear polymers and their corresponding SCNPs. Experiments were performed manually on a Bruker Advance III 400 MHz spectrometer using D_2O at 25°C and a polymer concentration of 5 mg/mL. Particle radii were estimated using the Stokes–Einstein equation.

$$R_H = {(k_B \times T)}/{6\pi\eta D}$$

Where:

- k_B: the Boltzmann constant
- T: temperature (K)
- η: liquid's viscosity
- D: the diffusion coefficient measured by DOSY NMR

Fitted function:	$e^{-D\times\gamma^2\times g^2\times\delta^2(\Delta-\delta/3-\tau/2)} \times 10^4$
used gamma:	26752 rad/(s*Gauss)
used little delta:	0.0060000 s
used big delta:	0.074900 s
used gradient strength:	variable
Random error estimation of data:	RMS per spectrum (or trace/plane)
Systematic error estimation of data:	worst case per peak scenario

Fit parameter Error estimation method:	from fit using arbitrary y uncertainties
Confidence level:	95%
Used integrals:	area integral
Used Gradient strength:	all values (including replicates) used

2. Synthesis

a. Synthesis of RAFT agent (M-CPP)

Methyl 4-cyano-4-(((propylthio)carbonothioyl)thio)pentanoate (M-CPP) was prepared as the previously reported.[1, 2]

Synthesis of 1-O-acryloyl-2,3:4,5-di-O-isopropylidene-β-D-fructopyranose (pFruA) monomer

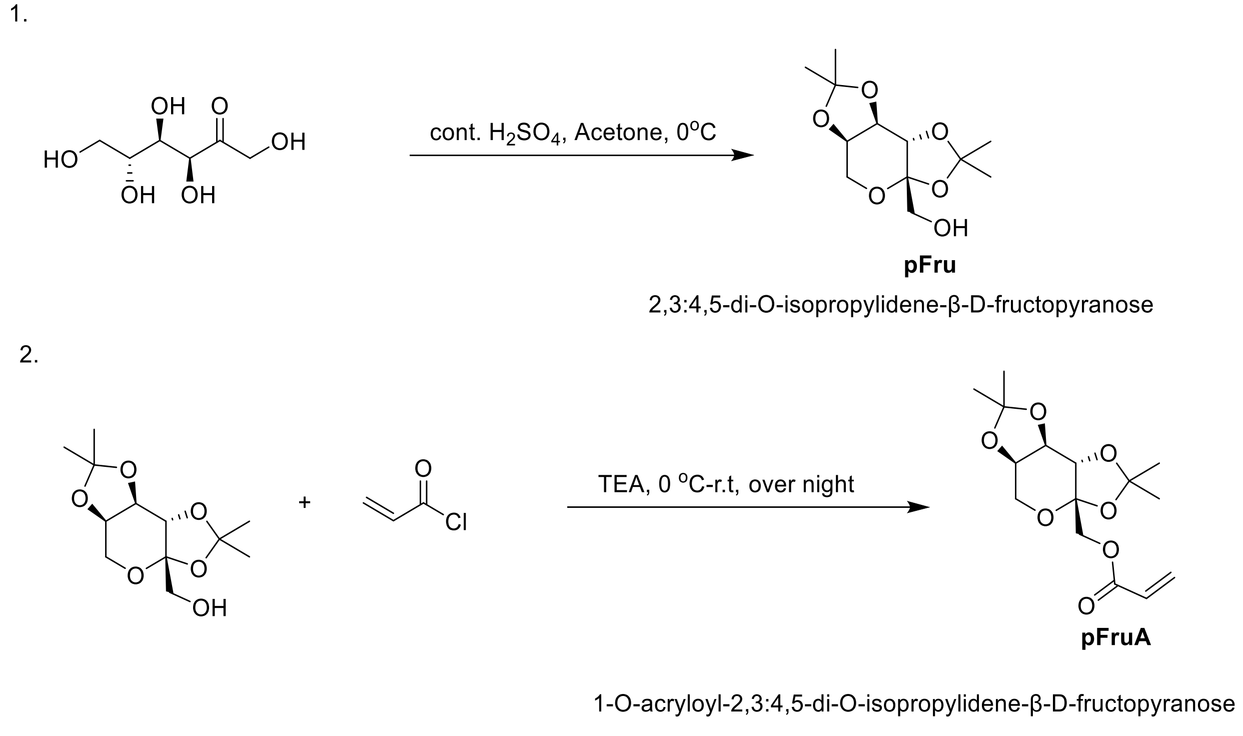

Scheme S1. Preparation of 2,3:4,5-di-O-isopropylidene-β-D-fructopyranose (pFru)

In a 2 L Erlenmeyer flask, d-fructose (36.0 g, 200 mmol, 1.0 eq.) was added to the prepared solution of cold concentrated sulfuric acid (35 mL) in acetone (500 mL). The suspension was stirred at

room temperature for 2 h before being cooled in an ice bath. Next, a cold solution of NaOH (110 g, 220 mmol, 1.1 eq.) in 500 mL of water was added gradually while stirring for 30 min. After that, acetone was removed by reduced pressure, and the crude product was extracted with DCM and washed with brine (3 × 200 mL) before being dried over Na_2SO_4. The solvent was removed by rotary evaporation, and the crude product was recrystallized twice in ether, yielding a white solid of pFru (70%).

^{1}H NMR (400 MHz, DMSO) δ 5.04 (dd, *J* = 6.9, 5.7 Hz, 1H, -OH), 4.57 (dd, *J* = 7.9, 2.5 Hz, 1H), 4.28 (d, *J* = 2.5 Hz, 1H), 4.22 (dq, *J* = 7.9, 0.8 Hz, 1H), 3.74 (dd, *J* = 13.0, 1.9 Hz, 1H), 3.54 (dd, *J* = 12.9, 0.8 Hz, 1H), 3.44 (dd, *J* = 11.7, 5.7 Hz, 1H), 3.36 (dd, *J* = 11.7, 7.0 Hz, 1H), 1.45 (s, 3H), 1.35 (d, *J* = 1.7 Hz, 6H), 1.28 (s, 3H).

^{13}C NMR (101 MHz, DMSO) δ 108.40, 107.93, 103.65, 70.84, 70.06, 69.44, 63.11, 60.68, 26.87, 26.17, 26.01, 24.43.

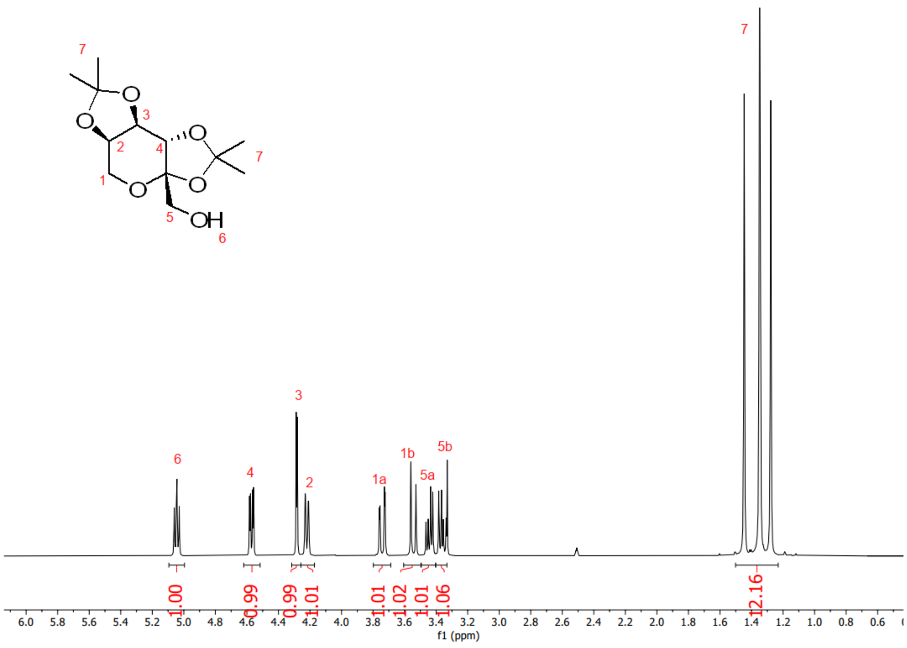

Figure S1. ^{1}H NMR spectrum of pFru in DMSO-d_6.

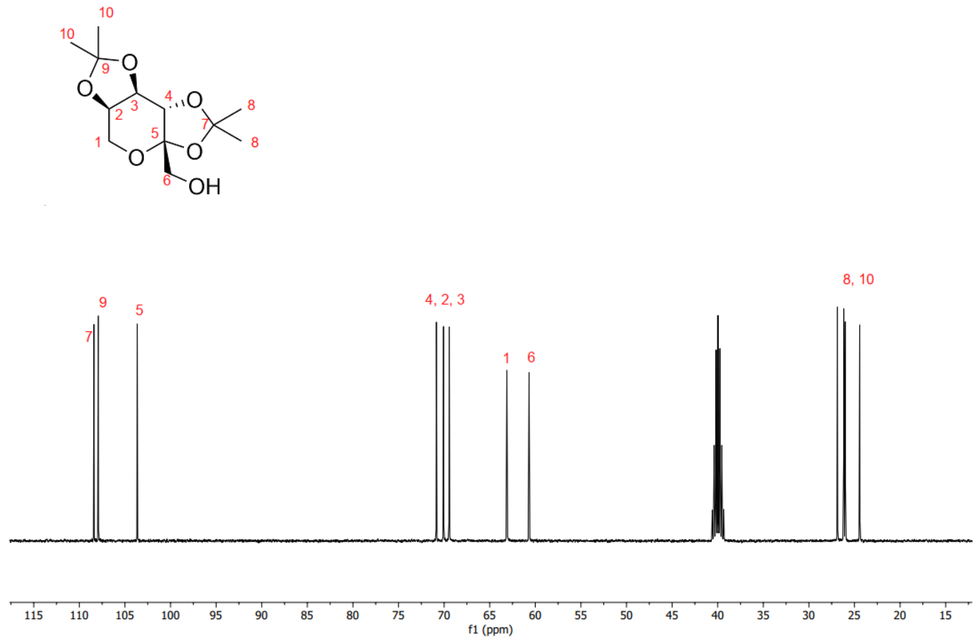

Figure S2. ^{13}C NMR spectrum of pFru in DMSO-d$_6$.

Synthesis of 1-O-acryloyl-2,3:4,5-di-O-isopropylidene-β-D-fructopyranose (pFruA):

In a 250 mL two-neck round-bottom flask, pFru (9.064 g, 34.8 mmol, 1 eq.) and triethylamine (TEA) (7.05 g, 69.6 mmol, 2 eq.) were dissolved in anhydrous DCM (150 mL), and the solution was cooled in an ice bath. Next, acryloyl chloride (4.73 g, 52.2 mmol, 1.5 eq.) in anhydrous DCM (50 mL) was added dropwise while stirring under N_2. After that, the solution was left to react overnight at room temperature. The reaction mixture was extracted with DCM, washed with brine (3 × 200 mL), and dried over Na_2SO_4. The solvent was removed, and the crude was purified by column chromatography on silica gel using ethyl acetate: n-hexane = 1:3 v/v % as the eluent, yielding transparent viscous oil, 70%.

^{1}H NMR (400 MHz, $CDCl_3$) δ 6.48 (dd, J = 17.3, 1.4 Hz, 1H), 6.18 (dd, J = 17.3, 10.4 Hz, 1H), 5.88 (dd, J = 10.4, 1.4 Hz, 1H), 4.63 (dd, J = 7.9, 2.6 Hz, 1H), 4.52 (d, J = 11.8 Hz, 1H), 4.37 (d, J = 2.6 Hz, 1H), 4.26 (ddd, J = 7.9, 1.9, 0.8 Hz, 1H), 4.15 (d, J = 11.7 Hz, 1H), 3.94 (dd, J = 13.0, 1.9 Hz, 1H), 3.79 (dd, J = 13.0, 0.8 Hz, 1H), 1.64 – 1.26 (m, 12H).

^{13}C NMR (101 MHz, $CDCl_3$) δ 165.45, 131.48, 128.01, 109.16, 108.78, 101.59, 70.78, 70.51, 70.08, 65.08, 61.28, 26.50, 25.92, 25.27, 24.05.

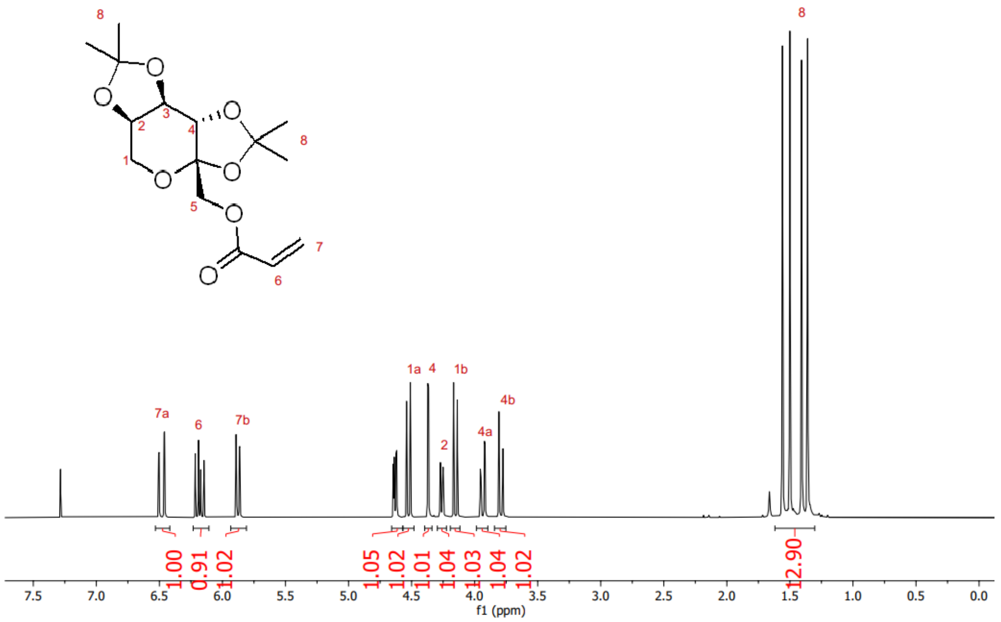

Figure S3. ^{1}H NMR spectrum of pFruA in CDCl₃.

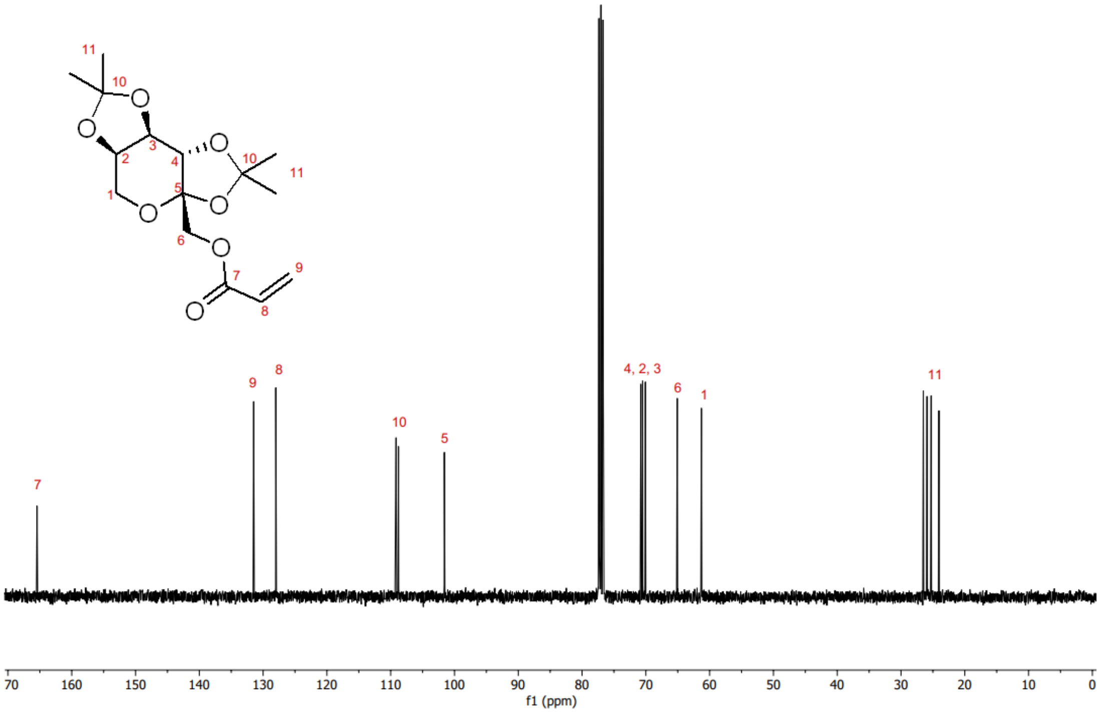

Figure S4. ^{13}C NMR spectrum of pFruA in CDCl₃.

b. Synthesis of polymer backbones

Library 1

Synthesis of polymer PEG0

In a 25 mL round-bottom flask, PEGMEA (4.8 g, 10 mmol), HEA (0.72 g, 6.2 mmol), M-CPP (0.055 g, 0.19 mmol), and AIBN (0.0046 g, 0.028 mmol) were dissolved in DMF (10 mL). Next, trioxane (0.1 g, 1.11 mmol) was added as an internal standard. The molar ratio of PEGMEA:HEA:M-CPP:AIBN was 53:33:1:0.15. The solution was degassed by bubbling with N_2 for 30 minutes before reacting at 70°C for 24 h. The crude was collected, precipitated in cold ether, and dried under a vacuum to yield a yellow liquid. The monomer conversion was >98% according to ^1H NMR.

Synthesis of polymer pPEG1

In a 10 mL round-bottom flask, polymer **PEG0** (1.4274 g, 0.05 mmol), pFruA (0.463 g, 1.47 mmol), and AIBN (0.0016 g, 0.01 mmol) were dissolved in DMF (5 mL). The molar ratio of pFruA:M-CPP:AIBN was 29.4:1:0.2. The solution was degassed by bubbling with N_2 for 20 minutes before reacting at 70°C for 40 h. The crude was collected and precipitated in cold ether and dried under a vacuum to yield a yellow liquid. The monomer conversion was >92% according to ^1H NMR.

Synthesis of polymer pPEG2

In a 10 mL round-bottom flask, polymer **PEG0** (1.1339 g, 0.039 mmol), pFruA (0.671 g, 2.13 mmol), and AIBN (0.0013 g, 0.008 mmol) were dissolved in DMF (5 mL). The molar ratio of pFruA:M-CPP:AIBN was 54.7:1:0.2. The solution was degassed by bubbling with N_2 for 20 minutes before reacting at 70°C for 40 hours. The crude was collected and precipitated in cold ether and dried under a vacuum to yield a yellow solid. The monomer conversion was >73% according to ^1H NMR.

Library 2

Synthesis of polymer pFru0

In a 25 mL round-bottom flask, pFruA (3.3 g, 10.44 mmol), HEA (0.78 g, 6.7 mmol), M-CPP (0.058 g, 0.2 mmol), and AIBN (0.0065 g, 0.04 mmol) were dissolved in DMF (10 mL). Next, trioxane (0.1 g, 1.11 mmol) was added as an internal standard. The molar ratio of pFruA:HEA:M-CPP:AIBN was 52:34:1:0.2. The solution was degassed by bubbling with N_2 for 30 min before reacting at 70°C for 24 h. The crude was collected and precipitated in cold ether and dried under a vacuum to yield a yellow solid. The monomer conversion was > 92% for pFruA and > 93% for HEA according to ^{1}H NMR.

Synthesis of polymer pFru1

In a 10 mL round-bottom flask, polymer **pFru0** (0.9837 g, 0.052 mmol), PEGMEA (0.712 g, 1.48 mmol), and AIBN (0.001 g, 0.01 mmol) were dissolved in DMF (5 mL). The molar ratio of PEGMEA:M-CPP:AIBN was 28.6:1:0.2. The solution was degassed by bubbling with N_2 for 20 min before reacting at 70°C for 24 h. The crude was collected and precipitated in cold diethyl ether, and dried under a vacuum to yield a yellow solid. The monomer conversion was > 84% according to ^{1}H NMR.

Synthesis of polymer pFru2

In a 10 mL round-bottom flask, polymer **pFru** (1.132 g, 0.06 mmol), PEGMEA (1.572 g, 3.28 mmol), and AIBN (0.002 g, 0.012 mmol) were dissolved in DMF (5 mL). The molar ratio of PEGMEA:M-CPP:AIBN was 55:1:0.2. The solution was degassed by bubbling with N_2 for 20 min before reacting at 70°C for 24 h. The crude was collected, precipitated in cold ether, and dried under a vacuum to yield a yellow solid. The monomer conversion was > 91% according to ^{1}H NMR.

Synthesis of polymer pFru3

In a 10 mL round-bottom flask, polymer **pFru0** (0.7482 g, 0.039 mmol), PEGMEA (2.06 g, 4.3 mmol), and AIBN (0.0013 g, 0.008 mmol) were dissolved in DMF (5 mL). The molar ratio of PEGMEA:M-CPP:AIBN was 109:1:0.2. The solution was degassed by bubbling with N_2 for 20 min before reacting at 70°C for 24 h. The crude was collected and precipitated in cold ether, dried under a vacuum to yield a yellow solid. The monomer conversion achieved was > 97% according to ^{1}H NMR.

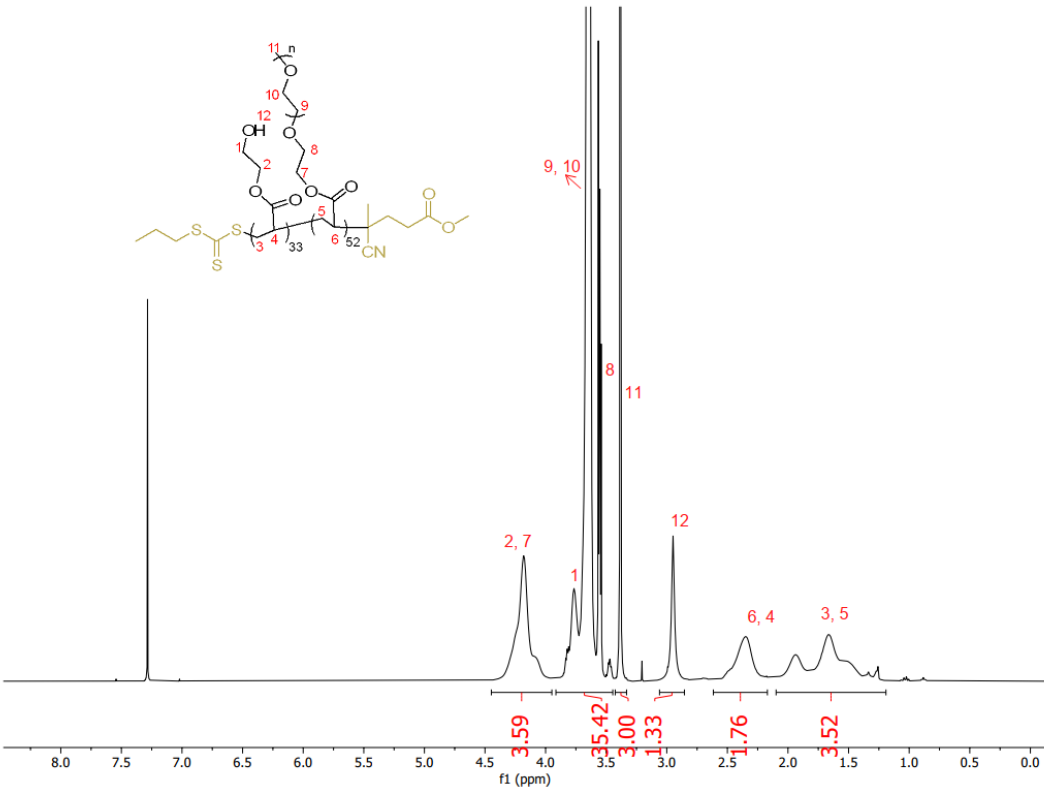

Figure S5. ^{1}H NMR spectrum of PEG0 in $CDCl_3$

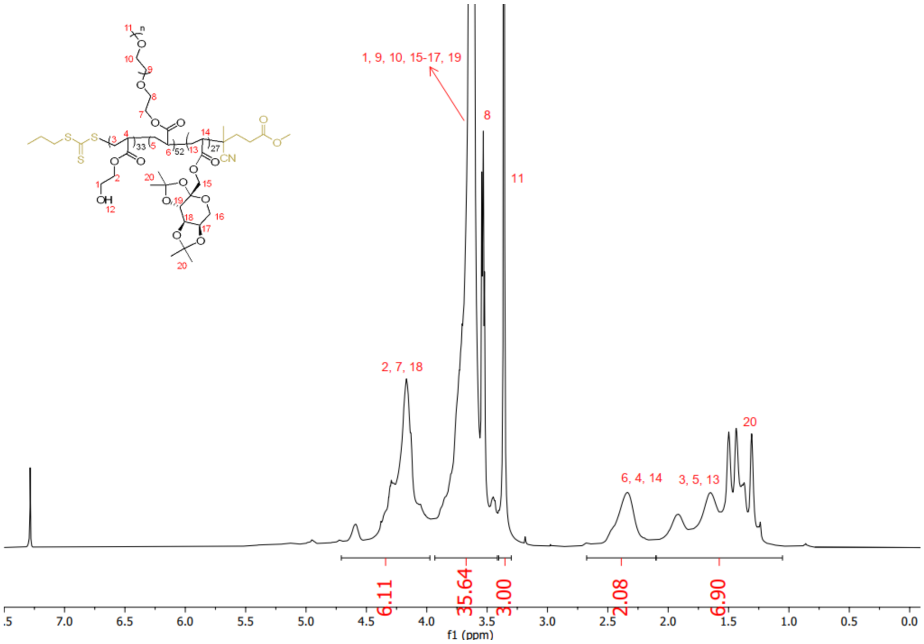

Figure S6. ^{1}H NMR spectrum of pPEG1 in $CDCl_3$

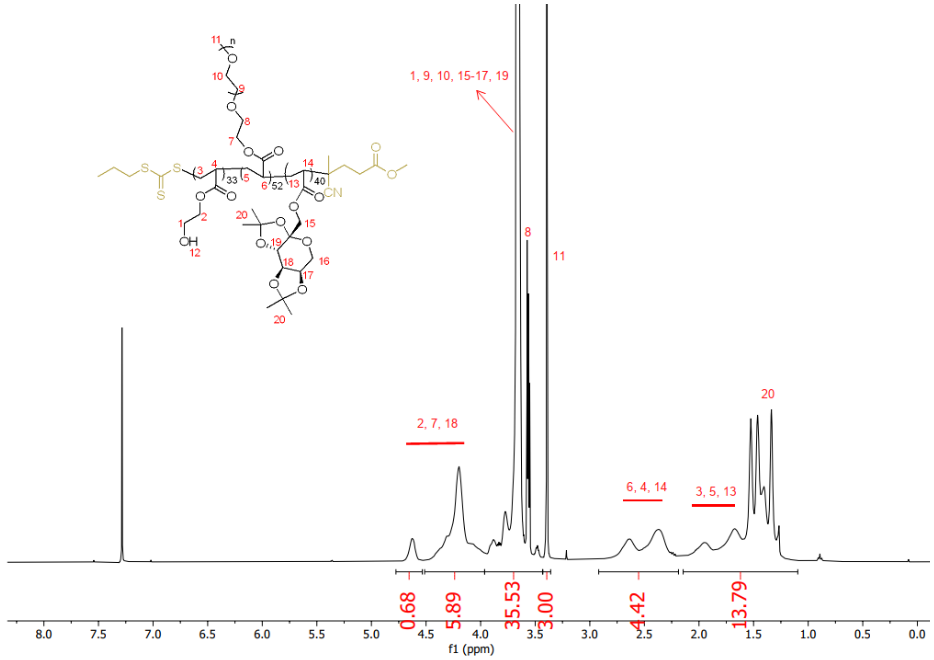

Figure S7. ^{1}H NMR spectrum of pPEG2 in $CDCl_3$

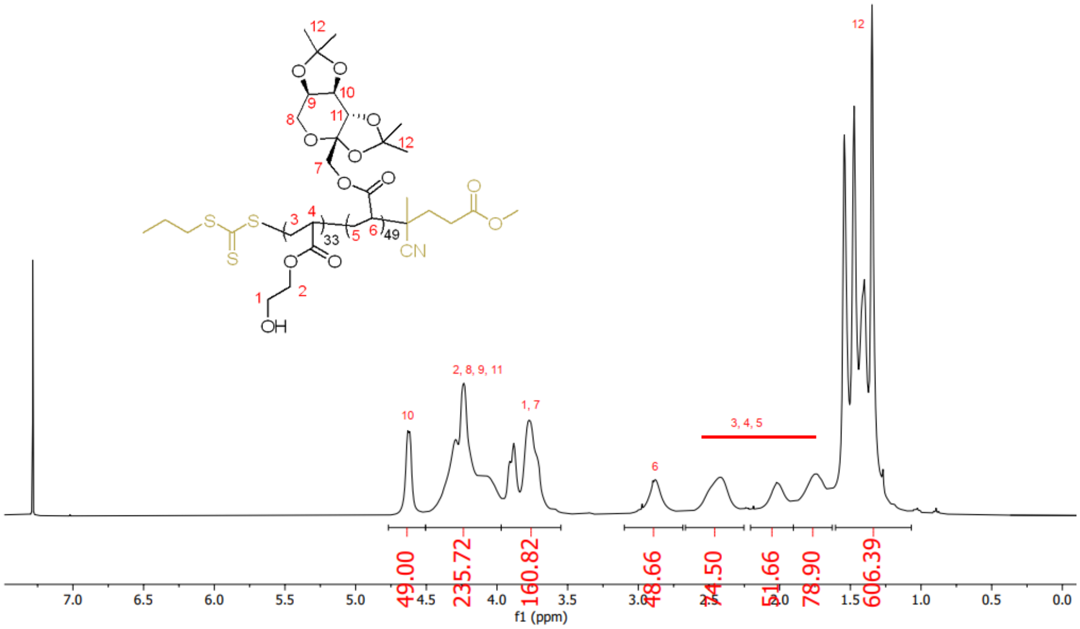

Figure S8. ^{1}H NMR spectrum of pFru0 in $CDCl_3$

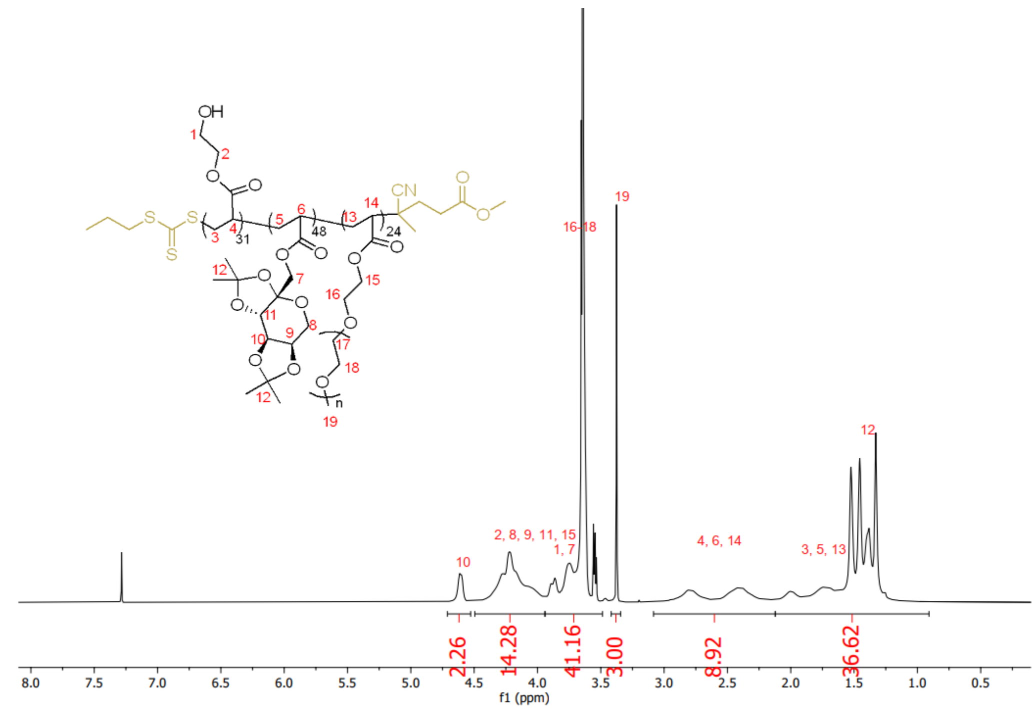

Figure S9. [1]*H NMR spectrum of pFru1 in* $CDCl_3$

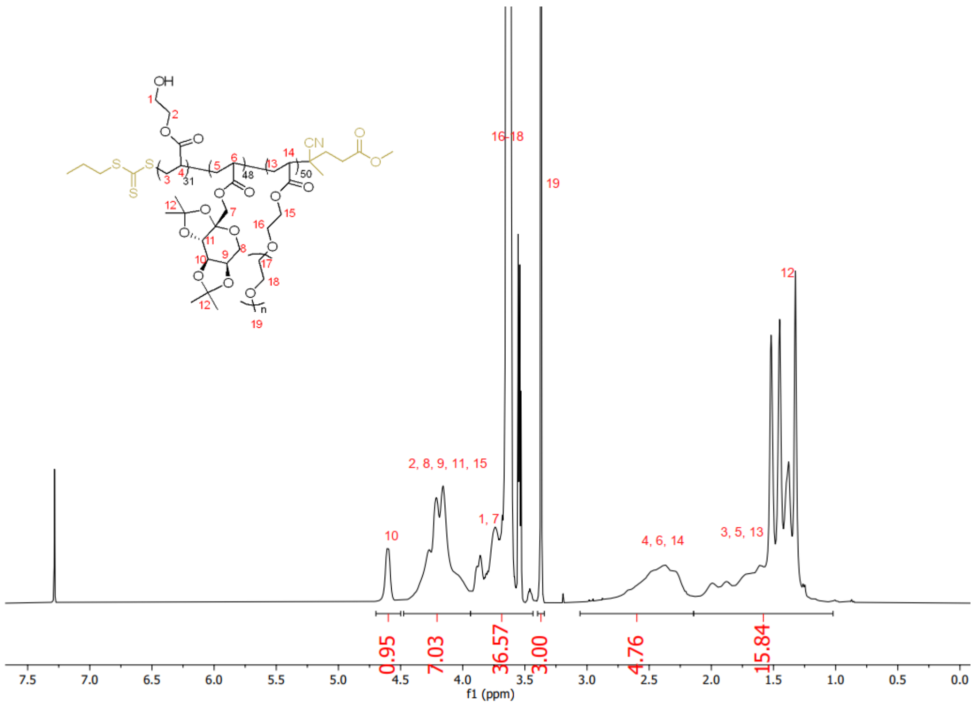

Figure S10. ^{1}H NMR spectrum of pFru2 in $CDCl_3$

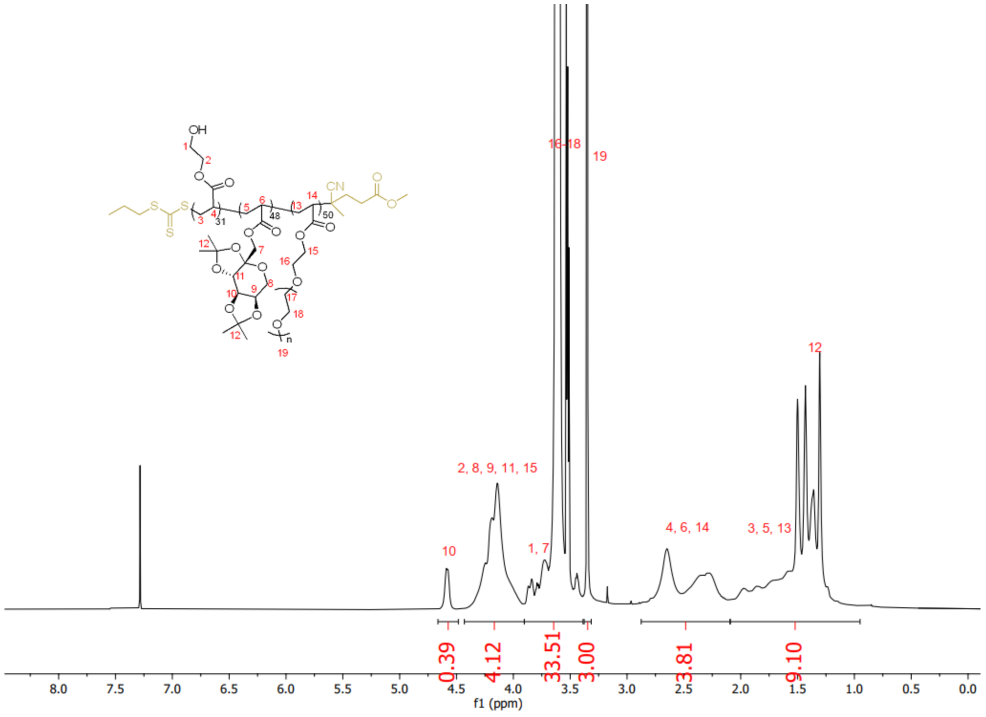

Figure S11. ^{1}H NMR spectrum of pFru3 in $CDCl_3$

c. Synthesis of the crosslinker

The crosslinker 4-(2-(2-methoxy-4-(2-(quinolin-2-yl)vinyl)phenoxy)ethoxy)-4-oxobutanoic acid (QIS) was prepared according to previous reports. [1, 2]

^{1}H NMR (400 MHz, DMSO) δ 8.33 (d, J = 8.6 Hz, 1H), 7.95 (ddd, J = 15.3, 8.4, 1.3 Hz, 2H), 7.85 (d, J = 8.6 Hz, 1H), 7.81 – 7.71 (m, 2H), 7.55 (ddd, J = 8.1, 6.9, 1.2 Hz, 1H), 7.45 – 7.37 (m, 2H), 7.25 (dd, J = 8.4, 2.0 Hz, 1H), 7.04 (d, J = 8.3 Hz, 1H), 4.37 (dd, J = 5.8, 3.3 Hz, 2H), 4.22 (dd, J = 5.5, 3.5 Hz, 2H), 3.88 (s, 3H), 2.57 (ddd, J = 7.1, 5.2, 1.5 Hz, 2H), 2.51 – 2.46 (m, 3H).

^{13}C NMR (101 MHz, DMSO) δ 173.85, 172.70, 156.42, 149.78, 148.91, 148.16, 136.85, 134.60, 130.27, 130.26, 128.99, 128.26, 127.36, 127.33, 126.44, 121.54, 120.21, 113.98, 110.61, 67.11, 63.20, 56.10, 29.09.

d. Conjugation of the crosslinker

The crosslinker was conjugated to the polymer *via* an EDC coupling reaction. The amount of crosslinker was calculated *via* ^{1}H NMR in $CDCl_3$ by the ratio of $-OCH_3$ group (δ ≈ 3.37 ppm) on

the polymer backbone and -CH of crosslinker (δ ≈ 6.88 ppm). For polymer pFru-HEA, the amount of crosslinker was calculated by comparison of the -CH group on the polymer backbone (δ ≈ 4.61 ppm) and -CH of crosslinker (δ ≈ 6.88 ppm).

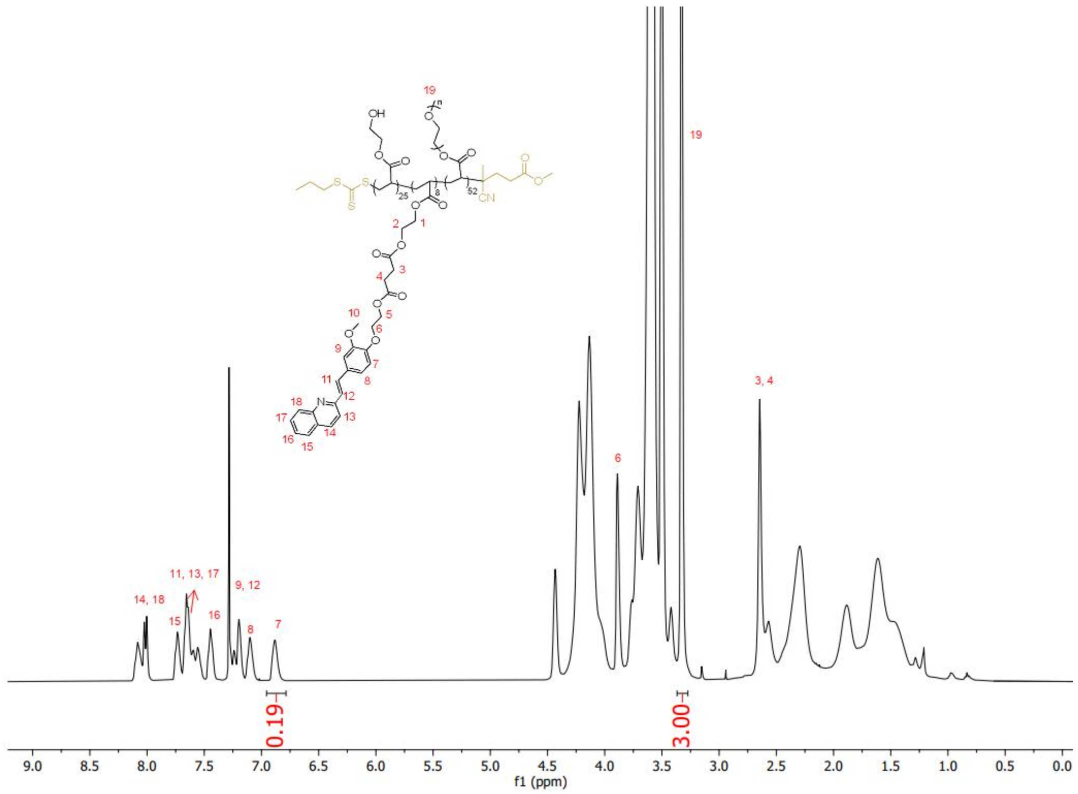

Figure S12. ^{1}H NMR spectrum of P-PEG0 in $CDCl_3$

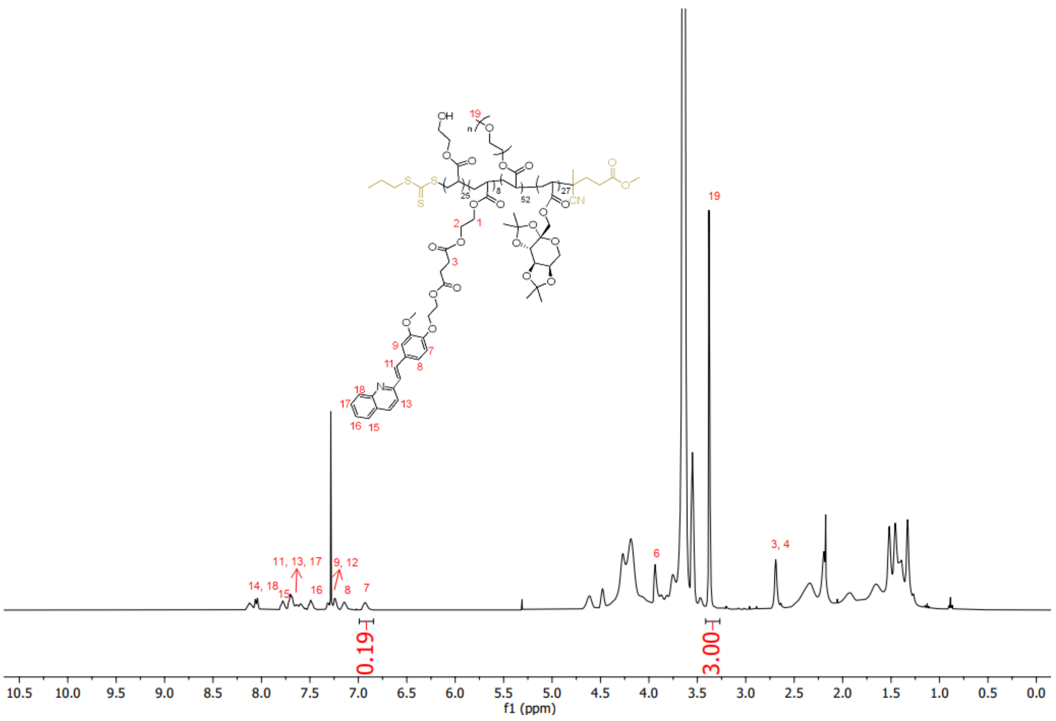

Figure S13. ^{1}H NMR spectrum of P-pPEG1 in $CDCl_3$

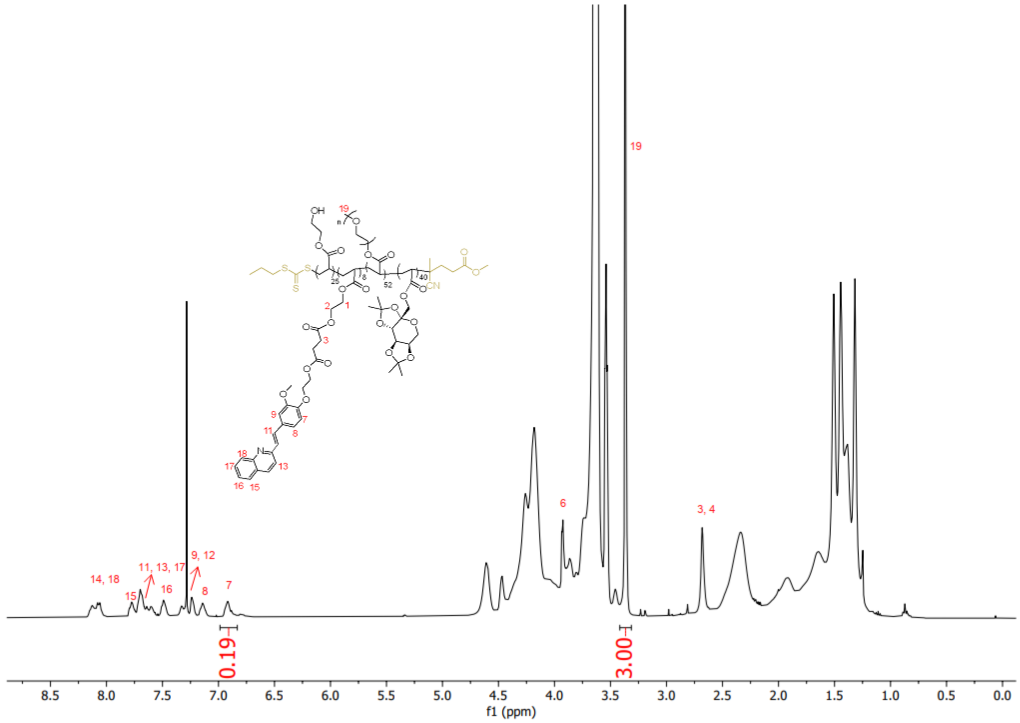

Figure S14. ^{1}H NMR spectrum of P-pPEG2 in $CDCl_3$

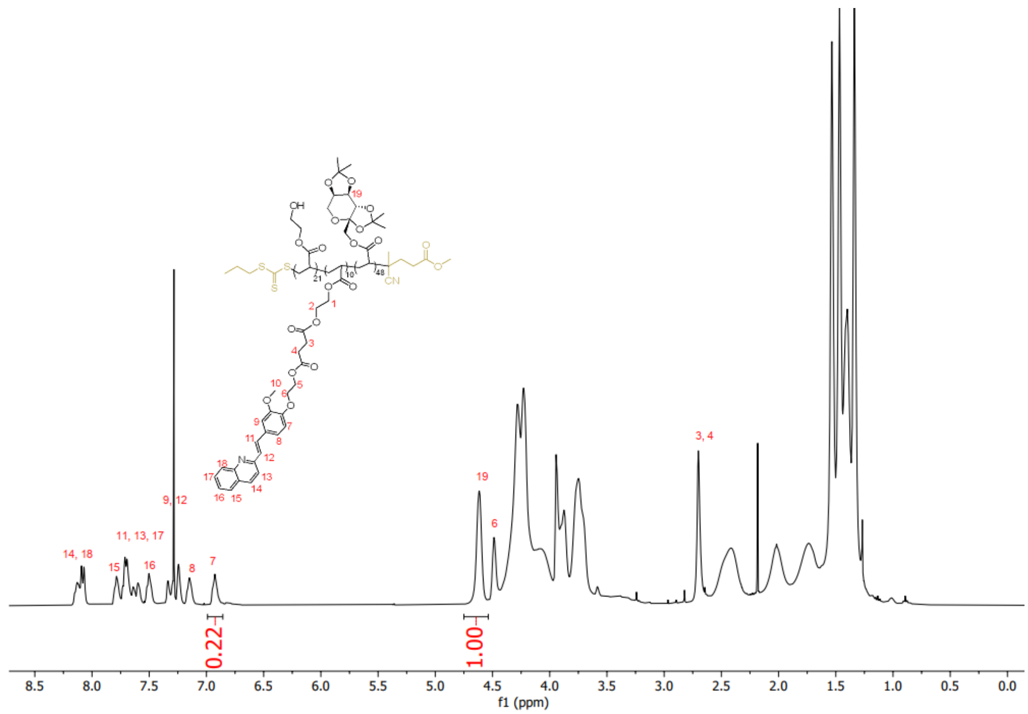

Figure S15. ^{1}H NMR spectrum of P-pFru0 in $CDCl_3$

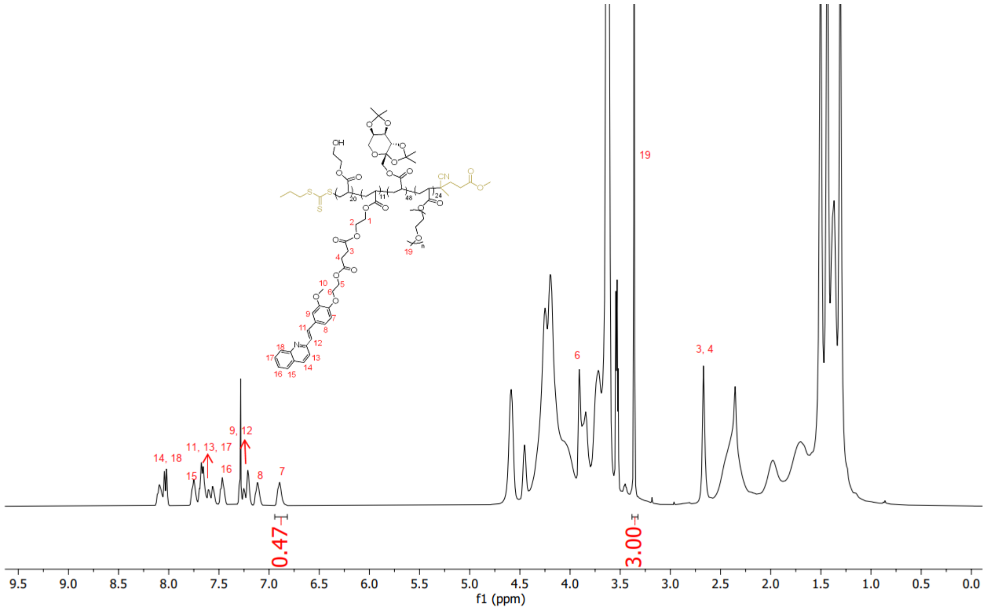

Figure S16. ^{1}H NMR spectrum of P-pFru1 in $CDCl_3$

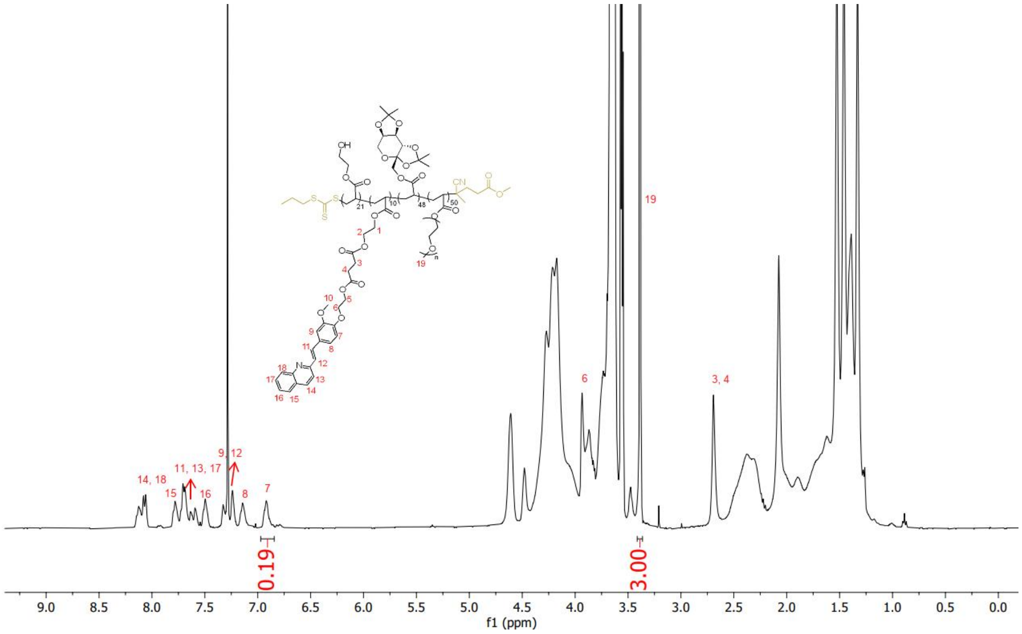

Figure S17. ^{1}H NMR spectrum of P-pFru2 in $CDCl_3$

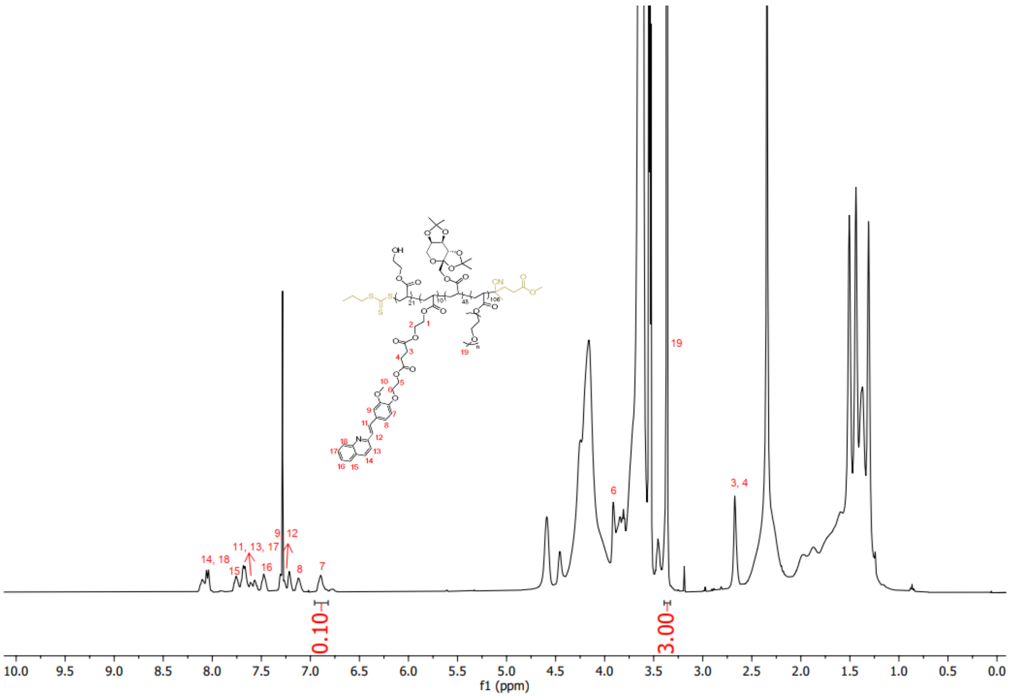

Figure S18. ^{1}H NMR spectrum of P-pFru3 in $CDCl_3$

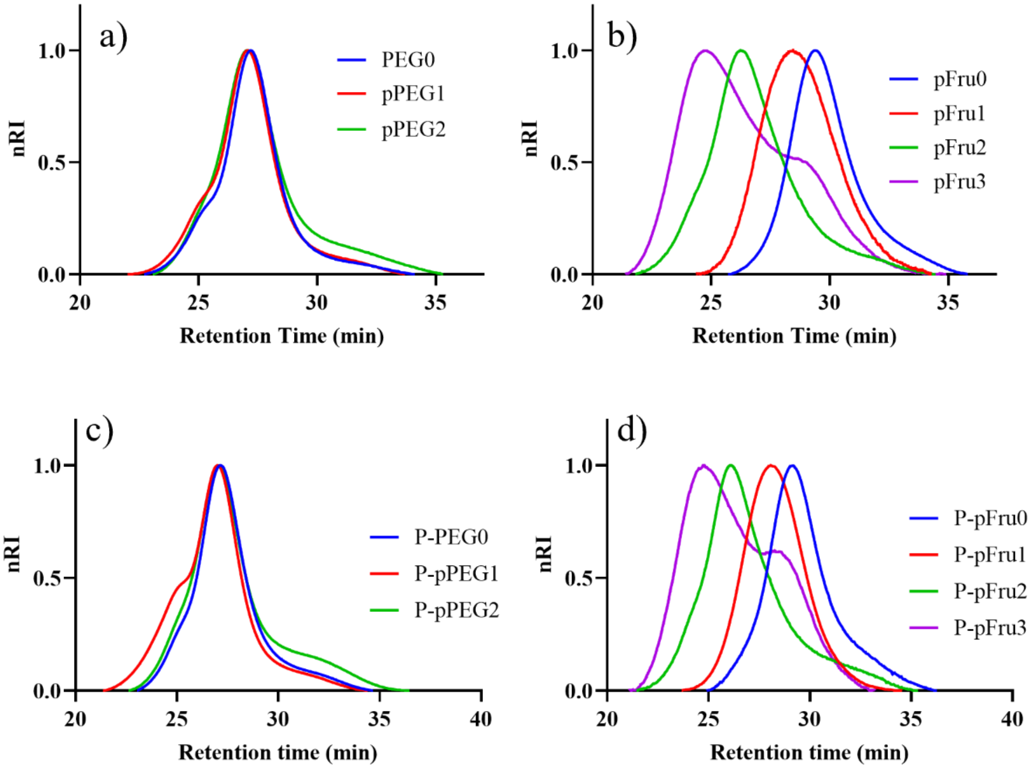

Figure S19. SEC traces in DMF of the polymer backbones (a) pPEG series and (b) pFruc series and their respective polymer-crosslinker conjugates (c and d).

Table S1. Molecular weight analysis of the polymer backbones and their corresponding conjugates with the crosslinker.

**Polymer**	**Structure**	**$M_{n,\ Theo}$[1] (g mol^{-1})**	**M_n[2] (g mol^{-1})**	**Đ[2]**
**PEG0**	PPEGMEA$_{52}$-*stat*-HEA$_{33}$	29000	22600	1.18
**pPEG1**	PPEGMEA$_{52}$-*stat*-HEA$_{33}$-*b*-pFru$_{27}$	37600	26800	1.17
**pPEG2**	PPEGMEA$_{52}$-*stat*-HEA$_{33}$-*b*-pFru$_{40}$	41700	20200	1.25
**pFru0**	PpFru$_{48}$-*stat*-HEA$_{31}$	19000	12900	1.12
**pFru1**	PpFru$_{48}$-*stat*-HEA$_{31}$-*b*-PEGMEA$_{24}$	30500	18700	1.19
**pFru2**	PpFru$_{48}$-*stat*-HEA$_{31}$-*b*-PEGMEA$_{50}$	43000	27200	1.28
**pFru3**	PpFru$_{48}$-*stat*-HEA$_{31}$-*b*-PEGMEA$_{106}$	69900	29600	1.43
**P-PEG0**	PPEGMEA$_{52}$-*stat*-HEA$_{25}$-*stat*-QIS$_{8}$	32300	21200	1.20

**P-pPEG1**	$PPEGMEA_{52}$-*stat*-HEA_{25}-*stat*-QIS_8-*b*-$pFru_{27}$	40800	27300	1.29
**P-pPEG2**	$PPEGMEA_{52}$-*stat*-HEA_{25}-*stat*-QIS_8-*b*-$pFru_{40}$	44900	18900	1.34
**P-pFru0**	$PpFru_{48}$-*stat*-HEA_{21}-*stat*-QIS_{10}	23000	13300	1.17
**P-pFru1**	$PpFru_{48}$-*stat*-HEA_{20}-*stat*-QIS_{11}-*b*-$PEGMEA_{24}$	29400	21400	1.14
**P-pFru2**	$PpFru_{48}$-*stat*-HEA_{21}-*stat*-QIS_{10}-*b*-$PEGMEA_{50}$	41700	22800	1.52
**P-pFru3**	$PpFru_{48}$-*stat*-HEA_{21}-*stat*-QIS_{10}-*b*-$PEGMEA_{106}$	68600	31200	1.37

[1]: estimated from ^{1}H NMR

[2]: measured from SEC traces in DMF

e. Deprotection of fructose

After crosslinker conjugation, the protecting groups of fructose moieties on the polymer were removed by treating the polymer-crosslinker conjugates with a TFA/H_2O mixture. In a 40 mL glass vial, P-pFru1 (1 g) was dissolved in DCM, (1 mL) then TFA/H_2O mixture (1.5 mL) at a ratio of 9:1 v/v was added dropwise. The solution was stirred overnight at room temperature. After that, the solvent and TFA were removed by stirring with an open cap for a day. The polymer was collected and dialyzed against water for 3 days. The solvent was removed by freeze drying, and the obtained polymer, after deprotection, was collected. ^{1}H NMR in $CDCl_3$ and DMSO-d_6 was used to confirm the successful deprotection by the disappearance of the peaks at $\delta \approx 1.34$-1.54 ppm.

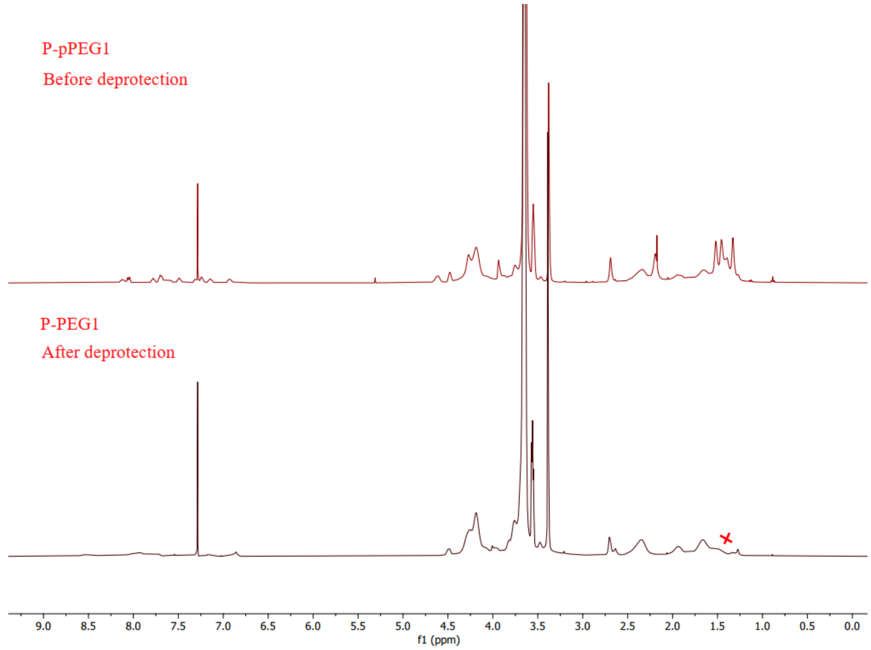

*Figure S20. *

Figure S20. ^{1}*H NMR spectra of copolymer in* $CDCl_3$ *before (P-pPEG1, upper) and after (P-PEG1, lower) deprotection.*

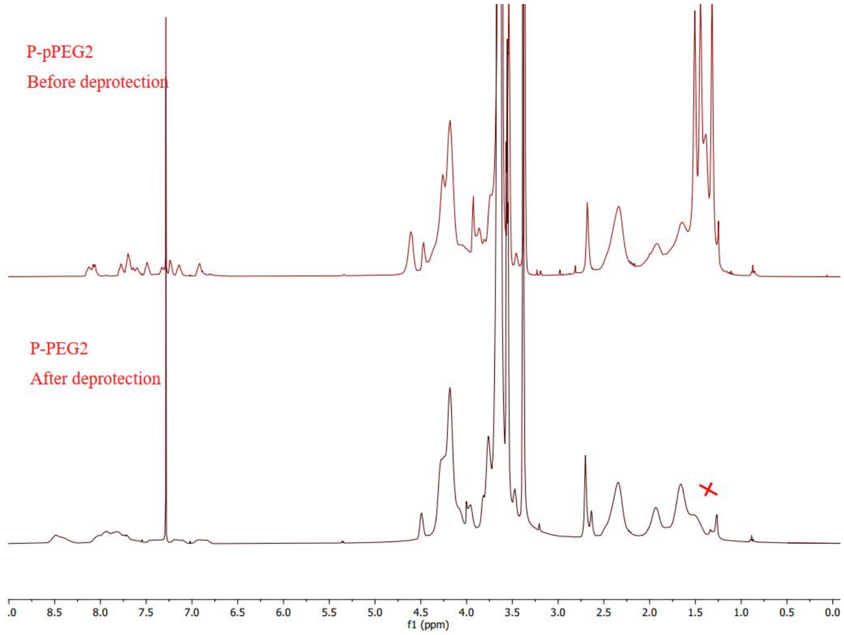

Figure S21. ^{1}*H NMR spectra of copolymer in* $CDCl_3$ *before (P-pPEG2, upper) and after (P-PEG2, lower) deprotection.*

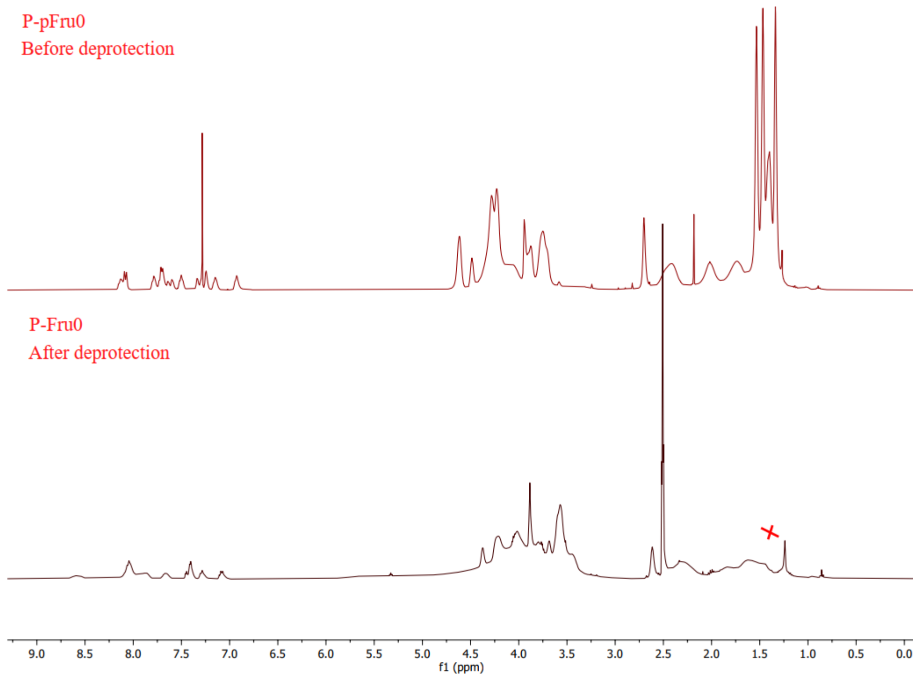

Figure S22. [1]*H NMR spectra of copolymer in* $CDCl_3$ *before (P-pFru0, upper) and in DMSO-*d_6 *after (P-Fru0, lower) deprotection.*

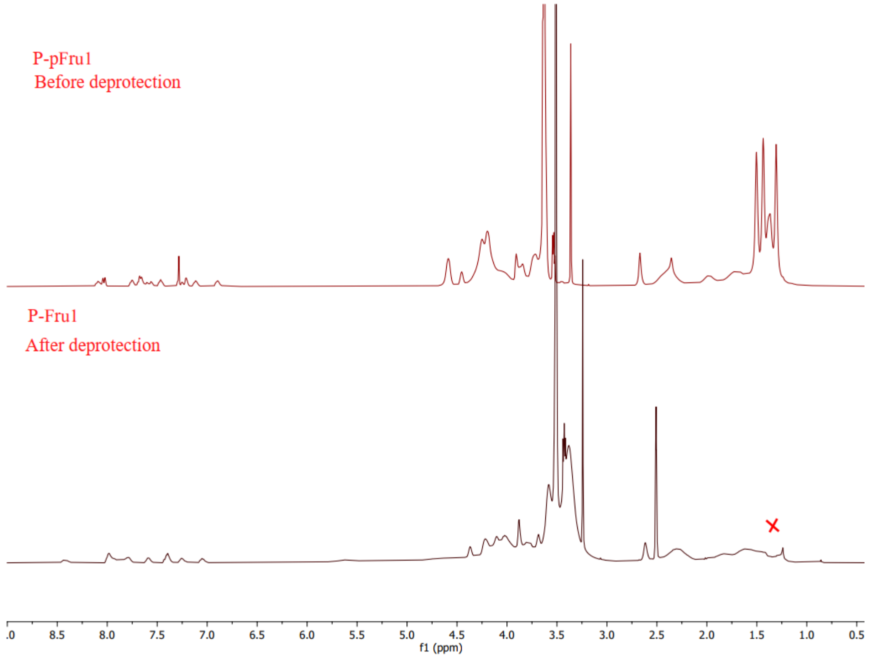

Figure S23. [1]*H NMR spectra of copolymer in* $CDCl_3$ *before (P-pFru1, upper) and in DMSO-*d_6 *after (P-Fru1, lower) deprotection.*

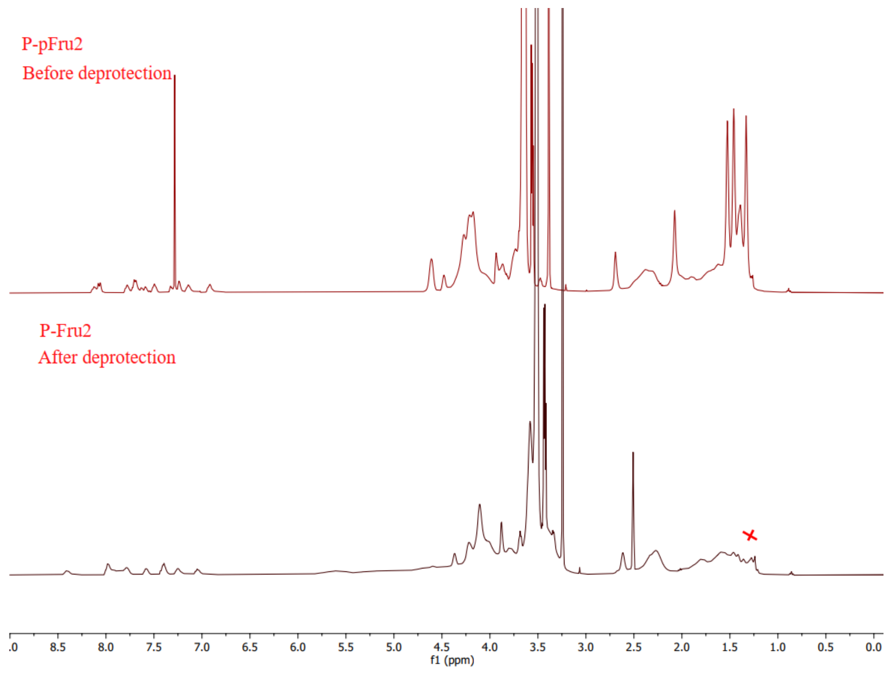

Figure S24. ^{1}H NMR spectra of the copolymer in $CDCl_3$ before (P-pFru2, upper) and in DMSO-d_6 after (P-Fru2, lower) deprotection.

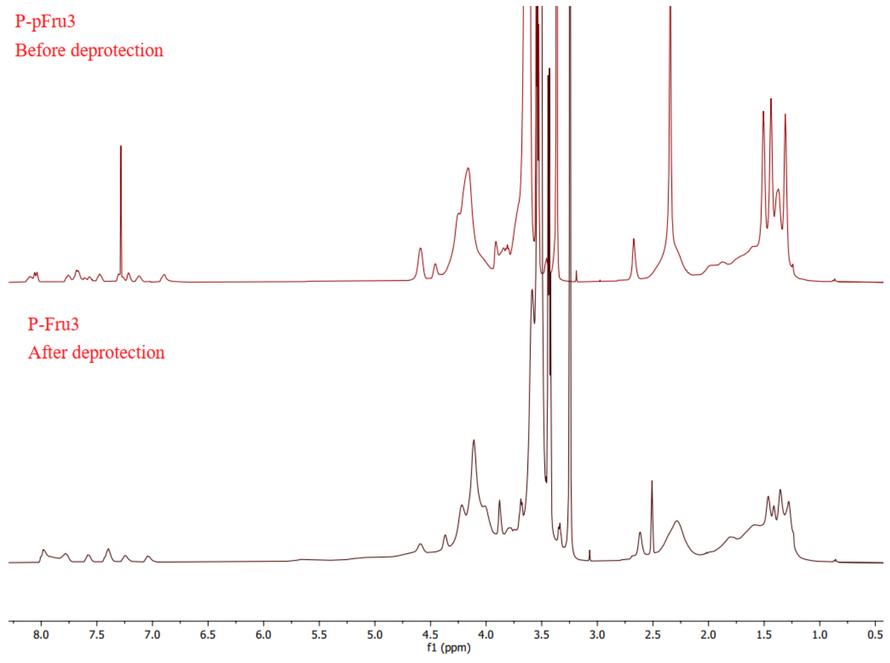

Figure S25. ^{1}H NMR spectra of the copolymer in $CDCl_3$ before (P-pFru3, upper) and in DMSO-d_6 after (P-Fru3, lower) deprotection. Due to the long chain PEG (106 units), the protecting groups on the sugar could not be removed completely.

f. SCNP preparation

For the preparation of SCNPs in water, a polymer was dissolved in Milli Q water at a set concentration and sonicated for 15 min. After that, the polymer solution was filtered through a 0.45 µm filter. Next, 1 mL of the solution was added to a 1.5 mL glass vial, placed in a custom-made irradiation box, and irradiated for the specified times. UV-Vis monitored the crosslinking, and the conversion was calculated by the reduction of the quinoline peak at $\lambda \approx 360$ nm over time.

Table S2. Optimization condition. Polymers were dissolved in water at a concentration of 1 mg mL^{-1}.

**Sample**	**Solvent**	**Concentration (mg/mL)**	**Irradiation time (min)**	**Conversion (%)**
**P-PEG0**	Water	1	5	40%
			**7**	**50%**
			10	74%
			20	76%
**P-PEG1**	Water	1	**5**	**51%**
			7	57%
			10	79%
			20	85%
**P-PEG2**	Water	1	**5**	**49%**
			7	63%
			10	82%
			20	86%
**P-Fru0**	Water	1	1	20%
			**1.5**	**49%**
			5	82%
			10	90%
**P-Fru1**	Water	1	2	36%
			**3**	**60%**
			5	80%

			10	87%
**P-Fru2**	Water	1	1	20%
			2	38%
			**3**	**67%**
			5	81%
			10	88%
**P-Fru3**	Water	1	1	26%
			**2**	**57%**
			2.5	65%
			5	87%

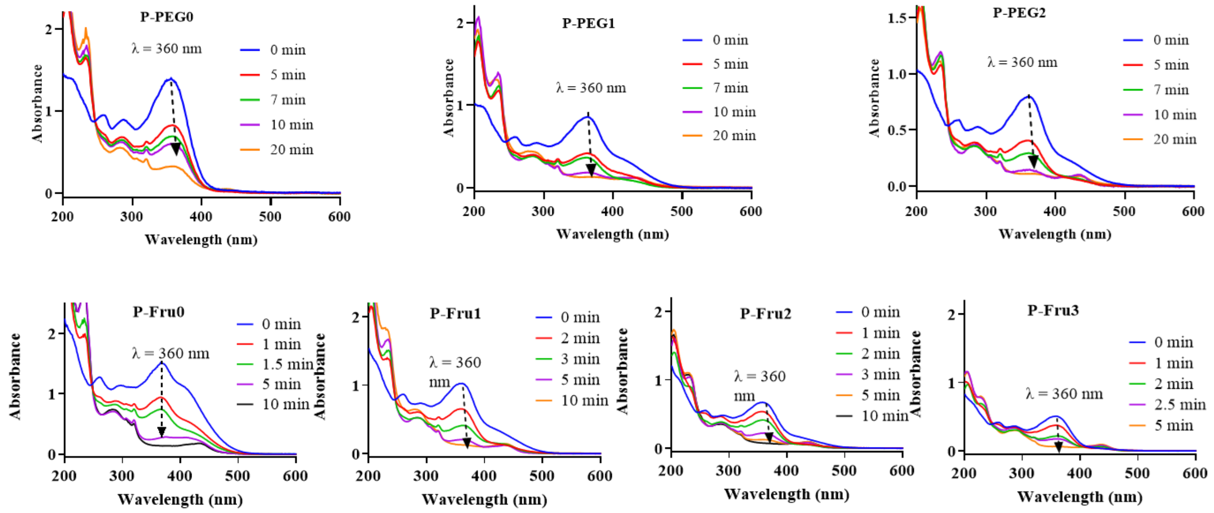

Figure S26. UV-Vis of polymer solution at 1 mg mL^{-1} in water, after different irradiation times.

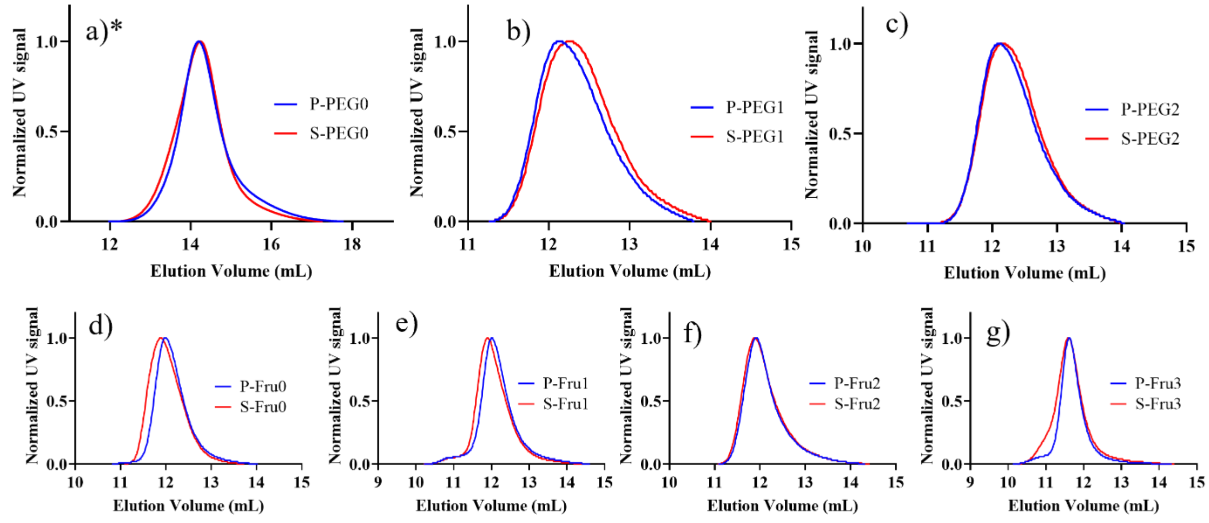

*Figure S27. SEC traces in an aqueous solution (Milli Q water + NaN_3) of the PEG-NPs (a-c) and Fru-NPs (d-g) before (blue) and after (red) crosslinking. * in 40% MeCN, 0.1% TFA in water.*

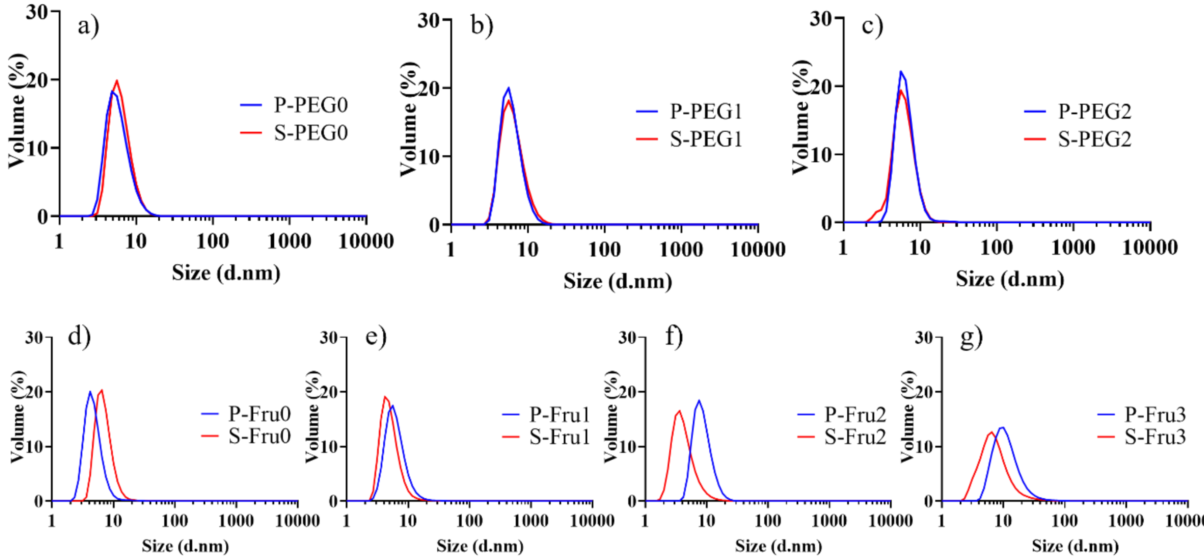

Figure S28. DLS measurements of polymer samples before (blue) and after (red) crosslinking of PEG-NPs (a-c) and Fru-NPs (d-g). Polymer solution at 1 mg mL^{-1} in water.

Table S3. Diffusion coefficients and hydrodynamic diameter of polymer before (P-X) and after (S-X) crosslinking calculated from DOSY-NMR. Polymer concentration at 5 mg mL $^{-1}$ in D_2O.

**Sample**	***D*** **[m^2 s^{-1}]**	***D_h*** **(nm)**
P-PEG0	6.7027E-11	5.96
S-PEG0	6.70434E-11	5.96
P-PEG1	5.85143E-11	6.83
S-PEG1	6.4073E-11	6.23
P-PEG2	5.851E-11	6.83
S-PEG2	6.60588E-11	6.05
P-Fru0	5.98582E-11	6.67
S-Fru0	6.33E-11	6.31
P-Fru1	7.62955E-11	5.24
S-Fru1	8.0868E-11	4.94
P-Fru2	4.84697E-11	8.24
S-Fru2	5.37026E-11	7.44
P-Fru3	4.87341E-11	8.20
S-Fru3	5.13089E-11	7.79

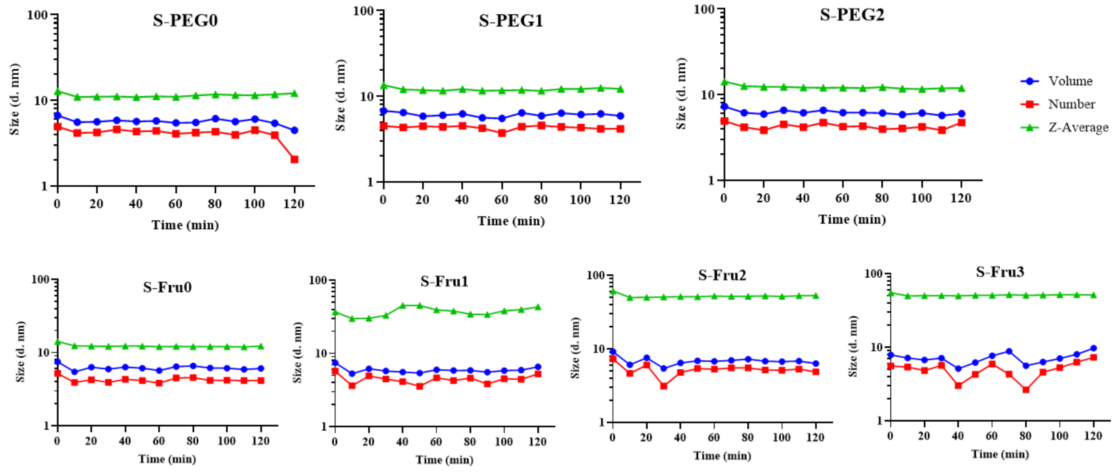

Figure S29. Colloidal stability of polymer nanoparticles in full DMEM at 37 °C over 2 h. The polymer concentration was 0.5 mg mL^{-1} in DMEM supplemented with 10% FBS, 1% penicillin, and 1% Glutamax. Measurements were performed at 37 °C and collected every 10 min.

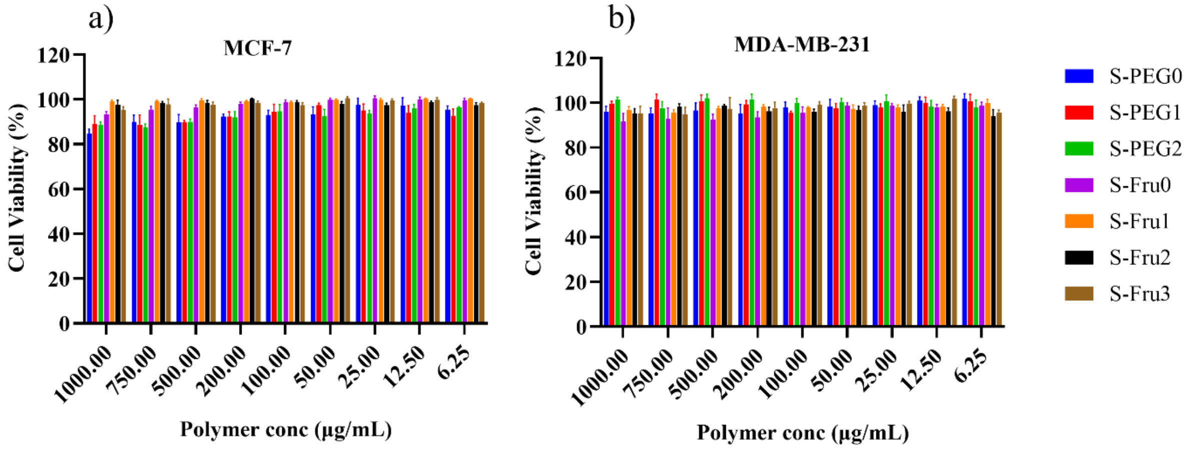

Figure S30. Cytotoxicity test of the polymer NPs on (a) MCF-7 and (b) MDA-MB-231 cells after 72 hours of incubation at various polymer concentrations.

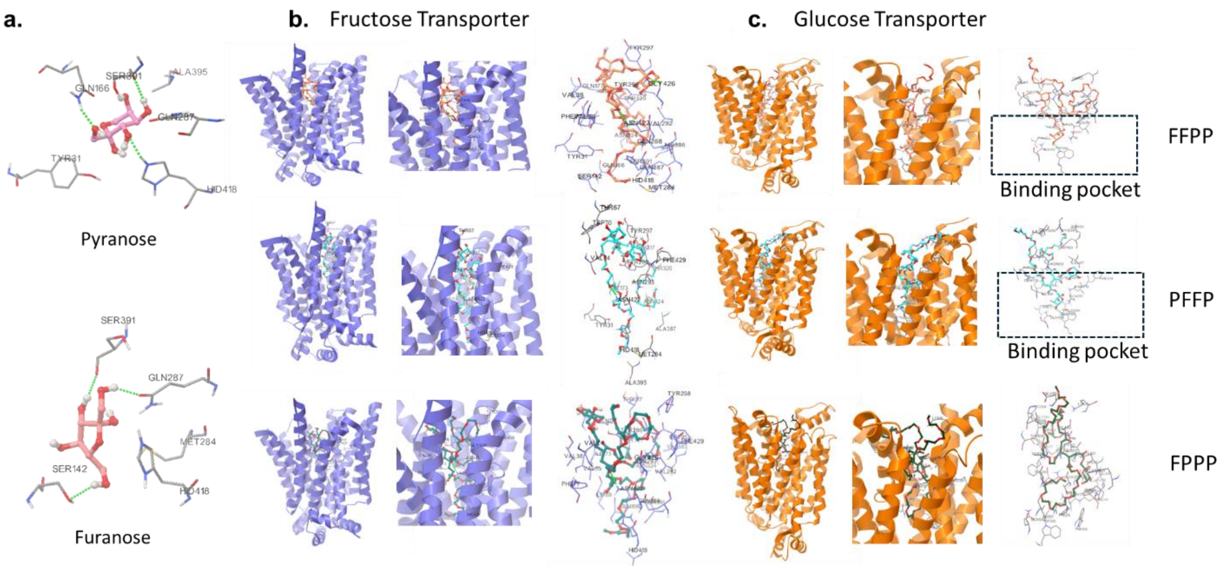

Figure S31. a.) interaction between the fructose transporter and fructopyranose or fructofuranose. The bindings between the polymer models and the fructose (b.) or glucose (c.) transporter are visualized, showing the overall protein-polymer complex, a zoomed-in view of the binding pocket, and detailed hydrogen bonding interactions, respectively. Hydrogen bonds are represented by green dashed lines.

g. Coupling of Cy5-COOH

For a typical procedure, S-Fru1 (0.059 g, 0.0019 mmol, 1 eq.) and EDC.HCl (0.0022 g, 0.0114 mmol, 6 eq.) in DCM (1 mL). Then, the prepared solution of Cy5-COOH (0.0012 g, 0.00228mmol, 1.2 eq.) and DMAP (0.0014 g, 0.0114mmol, 6 eq.) in DMF (1 mL) was added, and the solution

was stirred for 10 minutes. Next, the reaction solution was filled with nitrogen and reacted overnight at room temperature. After that, the polymer solution was collected and dialyzed against acetone for 2 days and water for 2 more days. The solvent was removed by freeze-drying, and the obtained polymer was collected. A similar procedure was applied for the coupling of Cy5 dye to the remaining SCNPs, keeping the same ratio of reactants.

References

1. Vo, Y.; Raveendran, R.; Cao, C.; Lai, R. Y.; Lossa, M.; Foster, H.; Stenzel, M. H., Solvent Choice during Flow Assembly of Photocross-Linked Single-Chain Nanoparticles and Micelles Affects Cellular Uptake. *ACS Applied Materials & Interfaces* **2024,** *16* (44), 59833-59848.

2. Vo, Y.; Raveendran, R.; Cao, C.; Tian, L.; Lai, R. Y.; Stenzel, M. H., Tadpole-like cationic single-chain nanoparticles display high cellular uptake. *Journal of Materials Chemistry B* **2024,** *12* (48), 12627-12640.
